# First discovery of triterpenoids and sterols from *Cotinus coggygria* var. *cinereus* Engl. with anti-inflammatory and antibacterial activities

**DOI:** 10.1007/s13659-025-00553-4

**Published:** 2026-01-05

**Authors:** Yue-Tong Zhu, Ze-Rui Li, Ren-Hao Chen, Jin-Hao Li, Wei Wang, Yu-Qi Gao, Chun-Huan Li, Jin-Ming Gao

**Affiliations:** 1 Shaanxi Key Laboratory of Natural Products & Chemical Biology, College of Chemistry & Pharmacy, Northwest A&F University, Yangling, 712100 People’s Republic of China; 2 College of Food Science and Technology, Northwest University, Xi’an, Shaanxi 710069 People’s Republic of China

**Keywords:** *Cotinus coggygria* var. *cinereus* Engl., Triterpenoids, Sterols, Anti-inflammatory activity, Antibacterial activity

## Abstract

This study led to the isolation of 17 triterpenoids, including two lanostane-type (**1** and **2**), two dammarane-type (**3** and **7**), ten tirucallane-type (**4** and **8**−**16**), and three oleanane-type (**17**−**19**) triterpenoids, as well as nine sterols (**5**, **6**, and **20**−**26**) from *Cotinus coggygria* var. cinereus Engl, which were first discovered from the genus *Cotinus*. Among them, coggygrenoids A−D (**1**−**4**), coggygrerol A (**5**), and coggygrerol B (**6**) are undescribed compounds. Additionally, seven flavonoids (**27**−**33**) were also isolated from this plant*.* Compound **15** displayed inhibitory activities in LPS-induced RAW 264.7 cells with an IC_50_ value 6.81 ± 0.15 μM. Molecular docking demonstrated that **15** exhibited favorable affinity for NLRP3 and iNOS. In vitro and in vivo antibacterial evaluations indicated that coggygrnoid C (**3**) exhibited significant inhibitory activity against methicillin-resistant *Staphylococcus aureus* ATCC BAA-1717 (USA300), with An MIC of 8 μg/mL. Further mechanistic investigations demonstrated that **3** exerted antibacterial activity by compromising the integrity of the cell wall and membrane. Notably, the combination of **3** with ampicillin exhibited an additive antibacterial effect. In the *Galleria mellonella* infection model, compound **3** exhibited comparable activity to that of the positive control at 20 mg/kg. These findings suggest that triterpenoids of *C. coggygria* are potential antibacterial lead agents.

**Graphical Abstract:**

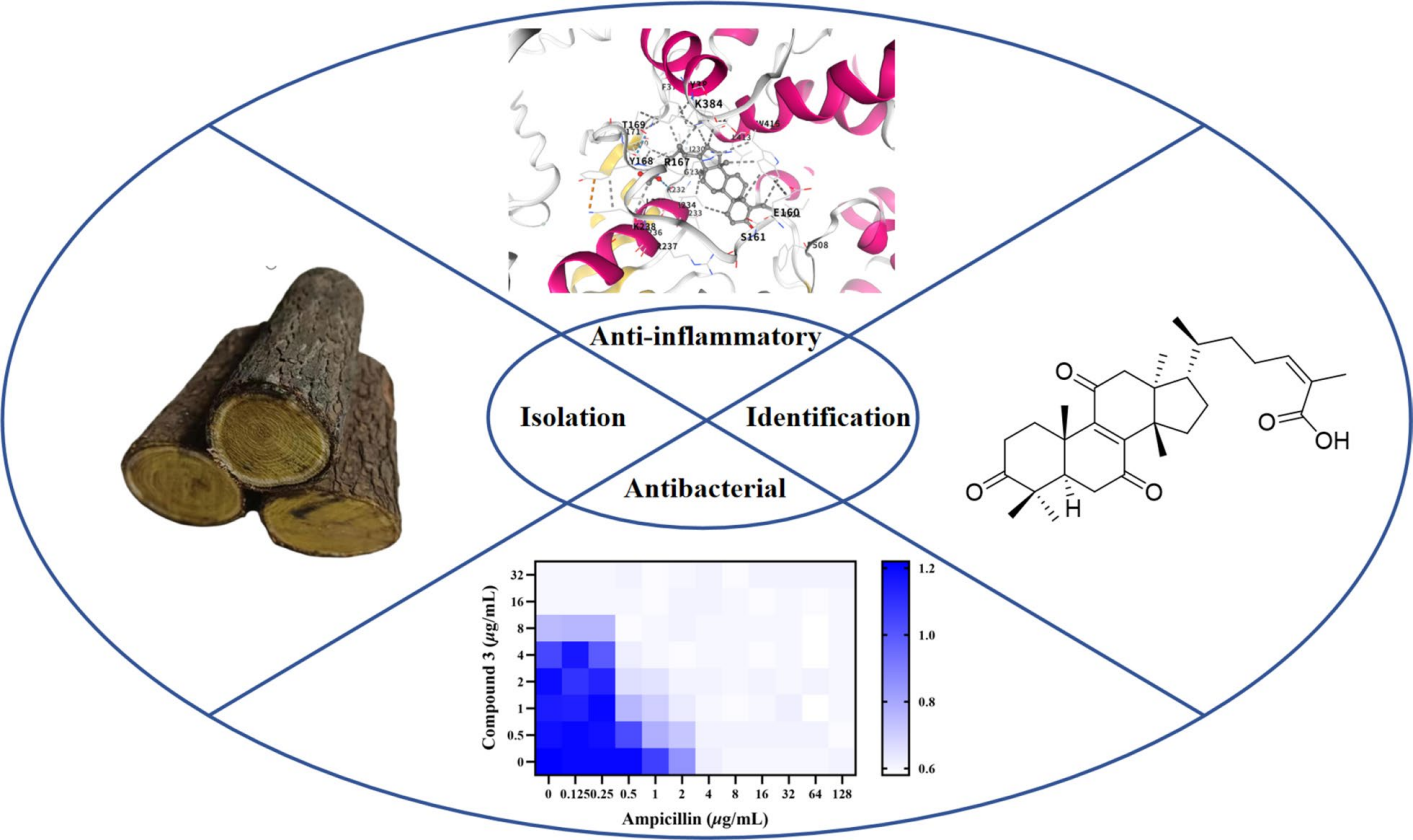

**Supplementary Information:**

The online version contains supplementary material available at 10.1007/s13659-025-00553-4.

## Introduction

*Cotinus* plants belong to the Anacardiaceae family and are categorized as deciduous shrubs or small trees [1]. These species are regarded as excellent ornamental tree species for landscaping purposes and served as pioneer tree species for afforestation on barren hills. *Cotinus* is rich in resources and widely distributed in Southern Europe, North America, China, and other regions. In China, *Cotinus* is primarily distributed in the mid-to-low altitude mountainous areas of Shaanxi, Shanxi, Henan, and Hebei provinces, with the main species being *Cotinus coggygria* var. *cinereus* Engl. [2]. *C. coggygria* contains a variety of chemical components, including flavonoids [3], gallic acid [4], and essential oils [5]. Historically, it has been extensively employed in traditional Chinese medicine and used for treating various diseases, including jaundice-type hepatitis, burns from fire or water, and lacquer dermatitis. Previous studies have shown that the extracts and chemical constituents of *C. coggygria* display extensive pharmacological effects, particularly in antioxidant [6, 7] hypotensive [8], liver damage repair [9], and antibacterial effects [10]. Triterpenoids are natural products composed of a basic nucleus consisting of six isoprene units totalling 30 carbon atoms, which have complex And diverse structures, encompassing over 100 different skeletal frameworks. A few of these are linear, monocyclic, bicyclic or tricyclic. The most common types are tetracyclic and pentacyclic triterpenes. Tetracyclic triterpenes are primarily classified as lanostane-, tirucallane-, dammarane- and cucurbitane-type. As important plant secondary metabolites, triterpenoids are involved in various physiological processes, such as oxidative stress, and exhibit pharmacological effects, including anti-inflammatory, neuroprotective, anti-Alzheimer's disease, antifungal and antitumour activities. As far as we know, no studies have been reported on the triterpenoids along with their biological activities in *C. coggygria*.

As part of an ongoing program of research into the identification of novel bioactive substances from traditional medicinal plants in the Tsinling Mountains [11–17], a phytochemical investigation was conducted on the tree stems and bark of *C. coggygria*. This investigation resulted in the isolation of a series of triterpenoids and sterols from the plant for the first time. The structures of the previously undescribed triterpenoids and sterols were elucidated using HRESIMS, NMR spectroscopy, and electronic circular dichroism (ECD) calculations. Furthermore, all isolates were evaluated for their anti-inflammatory activity in lipopolysaccharide (LPS) stimulated nitric oxide (NO) production in RAW 264.7 cells and their antibacterial activity against five gram-positive and five gram-negative bacteria. Herein, we describe the isolation, structural elucidation, biological activities, and mechanism of action of the isolated triterpenoids and sterols.

## Results and discussion

### Structure elucidation

**Fig. 1:**
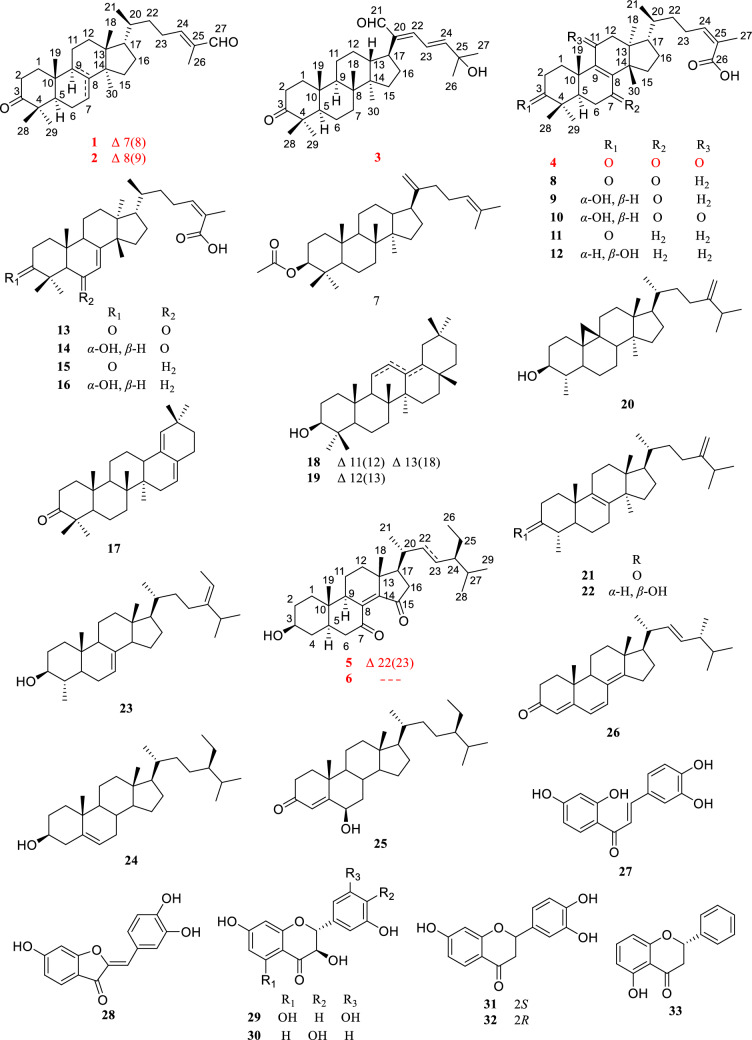
Structures of compounds **1**–**33**

**Fig. 2:**
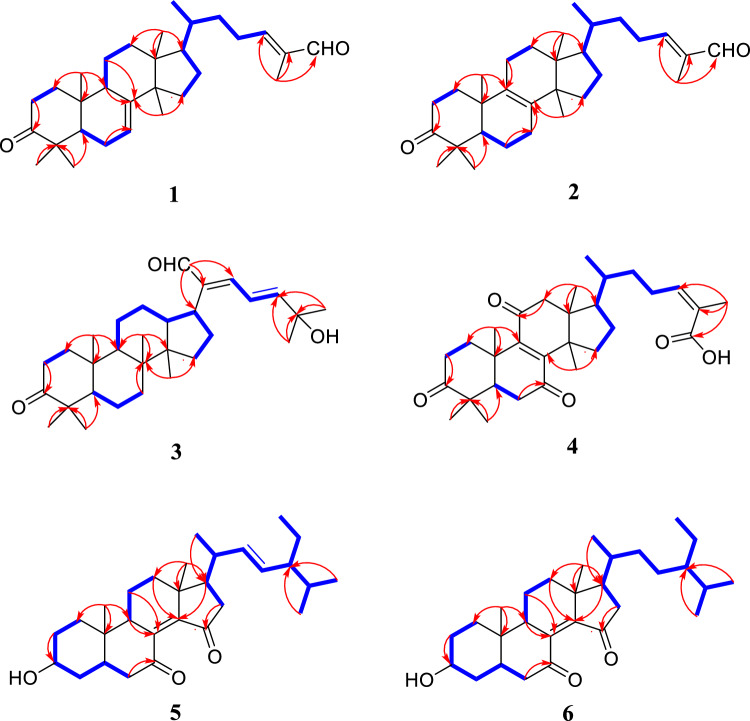
Key HMBC and ^1^H-^1^H COSY correlations of compounds **1**–**6**

**Fig. 3:**
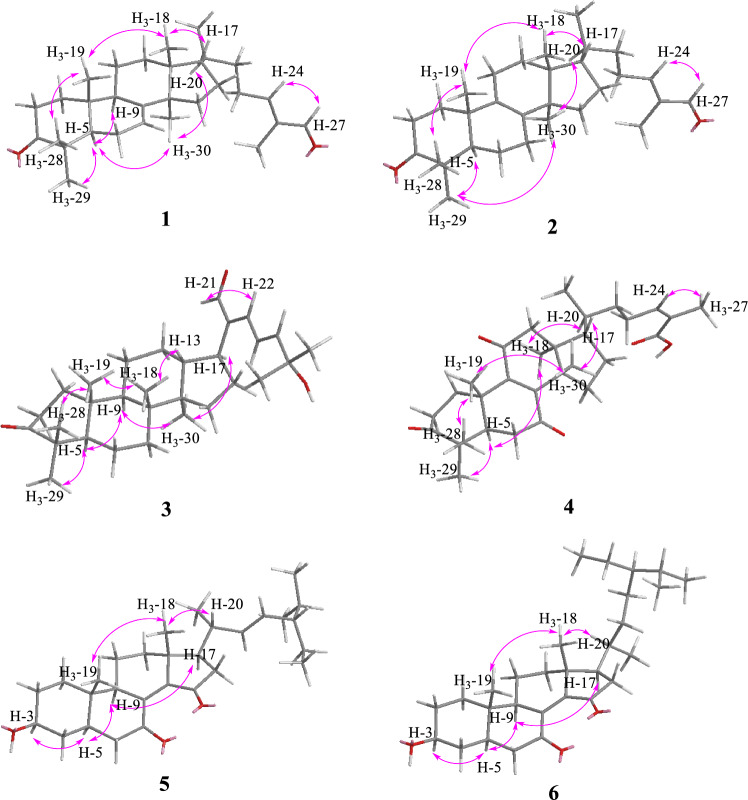
Key NOESY correlations of compounds **1**–**6**

**Fig. 4:**
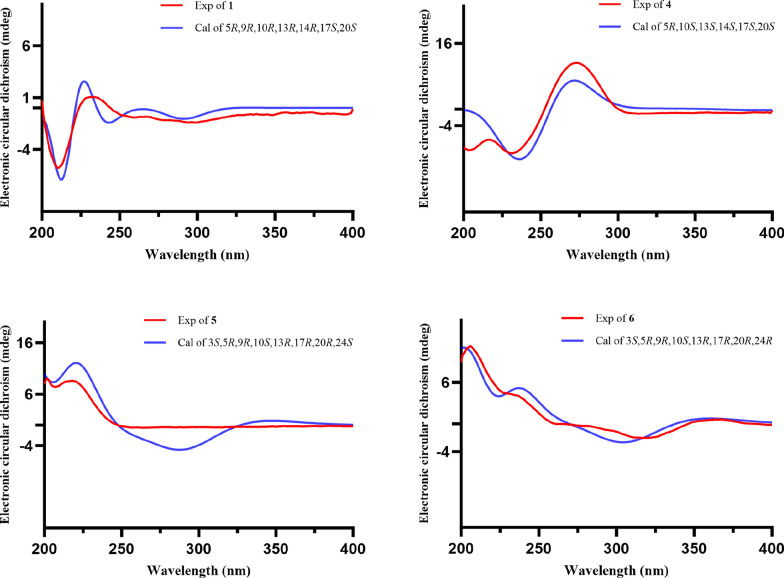
Experimental and calculated ECD curves of compounds **1** and **4**–**6**

Coggygrnoid A (**1**) was obtained as a colorless solid with the molecular formula C_30_H_46_O_2_, as established by a [M + Na]^+^ ion peak at *m/z* 461.3382 in its HRESIMS spectrum (calcd for C_30_H_46_O_2_Na, 461.3390), implying 11 degrees of unsaturation. The ^1^H NMR data (Table 1) of **1** indicated the presence of one aldehyde proton [*δ*_H_ 9.39 (1H, s, H-27)] and two olefinic protons [*δ*_H_ 6.49 (1H, t, *J* = 7.4 Hz, H-24), 5.31 (1H, m, H-7)]. In the high field, six tertiary methyl groups [*δ*_H_ 1.75 (3H, s, H_3_-26), 1.11 (3H, s, H_3_-29), 1.04 (3H, s, H_3_-28), 1.02 (3H, s, H_3_-30), 1.00 (3H, s, H_3_-19), 0.81 (3H, s, H_3_-18)], and a secondary methyl group [0.91 (3H, d, *J* = 6.1 Hz, H_3_-21)] were detected. The ^13^C NMR and HSQC spectra of **1** (Table 1) showed thirty carbon signals sorted as one keto-carbonyl [*δ*_C_ 217.0 (C-3)], one aldehyde [*δ*_C_ 195.5 (C-27)], two double bonds [*δ*_C_ 118.2 (C-7), 145.7 (C-8), 155.4 (C-24), 139.2 (C-25)], four quaternary carbons [*δ*_C_ 48.0 (C-4), 35.1 (C-10), 43.6 (C-13), 51.4 (C-14)], four methines [*δ*_C_ 52.4 (C-5), 48.5 (C-9), 53.3 (C-17), 36.0 (C-20)], nine methylenes [*δ*_C_ 38.6 (C-1), 35.1 (C-2), 24.5 (C-6), 18.4 (C-11), 33.9 (C-12), 34.0 (C-15), 28.6 (C-16), 33.7 (C-22), 26.6 (C-23)], and seven methyls [*δ*_C_ 22.4 (C-18), 12.9 (C-19), 18.6 (C-21), 9.2 (C-26), 24.7 (C-28), 21.7 (C-29), 27.6 (C-30)]. Analysis of the above spectral data suggests that compound **1** is a lanostane triterpenoid with an *α*,*β*-unsaturated aldehyde structural fragment at the end of its side chain [18]. Further Analysis of the 2D NMR data of compound **1** led to the determination of its planar structure. The ^1^H-^1^H COSY correlations (Fig. 2) revealed the presence of four spin-coupling systems (H_2_-1/H_2_-2; H-5/H_2_-6/H-7; H-5/H_2_-11/H_2_-12; H_2_-15/H_2_-16/H-17/H-20/(H_3_-21)/H_2_-22/H_2_-23/H-24) in **1**. The HMBC correlations (Fig. 2) of H_3_-28/H_3_-29 with C-3, C-4, and C-5; H_3_-19 with C-1, C-9, and C-10; H-2 with C-3; H-6 with C-8; H_3_-18 with C-12, C-13, and C-17; H_3_-30 with C-8, C-14, and C-15; and H_3_-26 with C-24, C-25, and C-27 established the carbon connectivity of **1**, and the planar structure of compound **1** was then determined as (24*E*)-3-oxo-5*α*-lanosta-7,24-dien-27-al. The relative configuration of compound 1 was established by analysing its NOESY spectrum. The NOESY correlations (Fig. 3) observed between H_3_-19 and H_3_-18/H_3_-28, as well as between H_3_-18 and H-17 indicate a co-facial configuration, and assigned as *β*-orientation. Meanwhile, the NOESY correlations between H-5 and H-9/H_3_-29/H_3_-30 revealed that they were *α*-oriented. The double bond Δ^24,25^ was determined as *Z*-configuration by the NOE-correlation of CHO-27 with H-24. To confirm the absolute structure of **1**, experimental and calculated ECD spectra (Fig. 4) were obtained, which verified the structure of **1**, as shown in Fig. 1.

**Table 1:** ^1^H NMR (400 MHz) and ^13^C NMR (100 MHz) data of **1**–**4** in CDCl_3_

No	**1**		**2**		**3**		**4**	
	*δ*_H_	*δ*_C_	*δ*_H_	*δ*_C_	*δ*_H_	*δ*_C_	*δ*_H_	*δ*_C_
1	1.99 (m)	38.6	2.02 (m)	35.7	1.90 (m)	40.1	2.76 (m)	34.7
	1.44 (m)		1.62 (m)		1.42 (m)		1.50 (m)	
2	2.75 (m)	35.1	2.54 (m)	34.7	2.45 (m)	34.2	2.76 (m)	34.4
	2.25 (m)		2.46 (m)				2.42 (m)	
3		217.0		218.4		218.2		214.5
4		48.0		47.4		47.6		46.9
5	1.71 (m)	52.4	1.69 (m)	51.6	1.38 (m)	55.5	2.14 (m)	49.4
6	2.09 (m)	24.5	1.62 (m)	20.3	1.56 (m)	19.8	2.56 (m)	36.4
			1.46 (m)		1.46 (m)		2.47 (m)	
7	5.31 (m)	118.2	2.12 (m)	27.6	1.59 (m)	34.9		199.0
			1.96 (m)		1.36 (m)			
8		145.7		134.8		40.5		150.3
9	2.26 (m)	48.5		132.7	1.40 (m)	50.2		153.6
10		35.1		37.3		37.0		38.0
11	1.56 (m)	18.4	2.02 (m)	21.5	1.47 (m)	22.0		202.3
					1.17 (m)			
12	1.82 (m)	33.9	1.80 (m)	31.1	1.33 (m)	25.2	2.68 (d, 16.4)	51.3
	1.63 (m)		1.65 (m)		1.08 (m)		2.49 (d, 16.4)	
13		43.6		44.2	2.06 (m)	46.4		45.2
14		51.4		49.7		50.4		48.0
15	1.49 (m)	34.0	1.53 (m)	29.8	1.94 (m)	32.2	2.17 (m)	32.0
			1.25 (m)		1.22 (m)		1.69 (m)	
16	1.96 (m)	28.6	1.93 (m)	28.3	1.86 (m)	27.6	2.05 (m)	28.0
	1.30 (m)		1.32 (m)		1.76 (m)		1.38 (m)	
17	1.51 (m)	53.3	1.56 (m)	50.3	2.86 (m)	37.9	1.66 (m)	49.2
18	0.81 (s)	22.4	0.76 (s)	15.9	0.92 (s)	15.4	0.92 (s)	18.3
19	1.00 (s)	12.9	1.05 (s)	19.9	1.05 (s)	15.3	1.50 (s)	17.4
20	1.45 (m)	36.0	1.52 (m)	36.0		144.6	1.42 (m)	36.1
21	0.90 (d, 6.1)	18.6	0.90 (d, 6.5)	18.9	9.39 (s)	195.5	0.90 (d, 6.6)	18.2
22	1.74 (m)	33.7	1.76 (m)	33.9	6.77 (d, 13.9)	149.9	1.55 (m)	35.5
	1.18 (m)		1.30 (m)				1.16 (m)	
23	2.41 (m)	26.6	2.38 (m)	26.0	6.77 (q, 13.9)	121.8	2.57 (m)	26.8
	2.26 (m)		2.27 (m)				2.45 (m)	
24	6.49 (t, 7.4)	155.4	6.49 (t, 7.2)	155.6	6.30 (d, 13.9)	150.9	6.07 (t, 7.1)	146.8
25		139.2		139.2		71.3		126.2
26	1.75 (s)	9.2	1.75 (s)	9.4	1.40 (s)	29.8		172.4
27	9.39 (s)	195.5	9.39 (s)	195.6	1.40 (s)	29.8	1.92 (s)	20.7
28	1.04 (s)	24.7	1.09 (s)	26.0	1.08 (s)	26.9	1.13 (s)	25.5
29	1.11 (s)	21.7	1.05 (s)	21.2	1.04 (s)	21.1	1.15 (s)	21.4
30	1.02 (s)	27.6	0.90 (s)	24.4	0.92 (s)	16.2	1.10 (s)	24.1

Coggygrnoid B (**2**) possessed a molecular formula of C_30_H_46_O_2_ (eight degrees of unsaturation), as determined by the quasi-molecular ion peak 461.3395 [M + Na]^+^ (calcd for C_30_H_46_O_2_Na, 461.3390) in its HRESIMS spectrum. The characteristic signals of one aldehyde proton at *δ*_H_ 9.39 (1H, s, H-27), an olefinic proton at *δ*_H_ 6.49 (1H, t, *J* = 7.2 Hz, H-24), and seven methyl signals at *δ*_H_ 1.75 (3H, s, H_3_-26), 1.09 (3H, s, H_3_-28), 1.05 (3H, s, H_3_-29), 1.05 (3H, s, H_3_-19), 0.90 (3H, d, *J* = 6.5 Hz, H_3_-21), 0.90 (3H, s, H_3_-30), And 0.76 (3H, s, H_3_-18) were presented in the ^1^H NMR spectrum (Table 1). A total of 30 carbon resonances were well resolved in the ^13^C NMR spectrum (Table 1), which were ascribed to seven methyls, ten methylenes, five methines (including two sp2 carbons), and eight quaternary carbons (one of which was carbonyl). Furthermore, a comparison of the ^1^H and ^13^C NMR data of **2** with those of compound **1** revealed close similarities. The Major difference was that the double bond at Δ 7(8) in **1** appeared at Δ 8(9) in **2**, which was supported by the chemical shifts of C-8 (*δ*_C_ 134.8), and C-9 (*δ*_C_ 132.7) and key HMBC correlations (Fig. 2) from H_2_-7 to C-8 and from H_2_-11 to C-9. Based on the biosynthetic relationship, combined with the NOESY correlations (Fig. 3) of H_3_-18/H-17, H_3_-18/H_3_-19, H_3_-19/H_3_-28, H_3_-29/H-5, H_3_-30/H-5, and CHO-27/H-24, the absolute configuration of **2** was assigned to be the same as those of **1**. Although we did not obtain the CD curve of **2**, its specific optical rotation value, which was similar to that of **1**, also confirmed this assignment. Thus, the structure of compound **2** was elucidated as (24*E*)-3-oxo-5*α*-lanosta-8,24-dien-27-al.

Coggygrnoid C (**3**) was obtained as a faint yellowish gum and showed the molecular formula C_30_H_46_O_3_ based on the HRESIMS ion at *m/z* 477.3330 [M + Na]^+^ (calcd for C_30_H_46_O_3_Na, 477.3339). The ^13^C NMR and HSQC spectra (Table 1) showed 30 carbon resonances, which were classified as seven quaternary carbons (one ketone carbonyl and one olefinic), eight methines (one aldehyde and three olefinic), eight methylenes, and seven methyls by their chemical shifts and hybridization states. Combining the above data with the unsaturation of compound **3** indicates that it contains four rings in its structure. Additionally, the presence of one conjugated diene group [*δ*_H_ 6.77 (1H, d, *J* = 13.9 Hz, H-22), 6.77 (1H, q, *J* = 13.9 Hz, H-23), 6.30 (1H, d, *J* = 13.9 Hz, H-24)], and seven tertiary methyls [*δ*_H_ 1.40 (3H, s, H_3_-26), 1.40 (3H, s, H_3_-27), 1.08 (3H, s, H_3_-28), 1.05 (3H, s, H_3_-19), 1.04 (3H, s, H_3_-29), 0.92 (3H, s, H_3_-18), And 0.92 (3H, s, H_3_-30)] were evident from the ^1^H NMR data (Table 1). The above data indicated that compound **3** was based on a dammarane triterpenoid skeleton similar to that of aglaiabbreviatin E (**7**) [19], with the main difference being the structure of the side chains. The exact structure of **3** was deduced from the ^1^H-^1^H COSY and HMBC spectra (Fig. 2). The HMBC correlations arising from CHO-21 to C-17, C-20, and C-22; the gem-dimethyl protons (H_3_-26 and H_3_-27) to C-24, and C-25, and the spin system (H-22/H-23/H-24) indicated the presence of an *α*,*β*,*γ*,*δ*,-unsaturated aldehyde, which is consistent with the UV absorption curve. Compound **3** shared the same relative configuration as those of other dammarane-type triterpenoids. The NOESY correlations (Fig. 3) of H_3_-18/H-13, H_3_-18/H_3_-19, and H_3_-19/H_3_-28 indicated co-facial orientation, which was assigned as *β*. Also, the NOESY correlations (Fig. 3) of H_3_-29/H-5, H-9/H-5, H-9/H_3_-30, and H_3_-30/H-17 suggested that these were *α*-oriented. Therefore, the structure of compound **3** was elucidated as (21*E*,23*E*)-3-oxo-25-hydroxydammar-21-al-20,23-diene.

Coggygrnoid D (**4**) gave a molecular formula of C_30_H_42_O_5_ as determined by an HRESIMS [M−H]^−^ peak at 481.2957 (calcd for C_30_H_41_O_5_, 481.2959). The ^1^H NMR spectrum (Table 1) exhibited the presence of seven methyl signals, including one methyl doublet at *δ*_H_ 0.90 (d, *J* = 6.6 Hz), five tertiary methyls, and one singlet methyl at *δ*_H_ 1.92. In addition, one olefinic proton was observed at *δ*_H_ 6.07 (t,* J* = 7.1 Hz). The ^13^C NMR spectrum (Table 1) displayed 30 carbon resonances. These were classified by HSQC experiment as three ketone carbonyls, one carboxyl group, two double bonds, four *sp*^3^ quaternary carbons, three *sp*^3^ methines, eight *sp*^3^ methylenes, and seven methyls in the structure. Comparing the NMR data of **4** with 3,7-dioxo-8,24*Z*-tirucalladien-26-oic acid (**8**) revealed that the structures were closely related. The only difference was the presence of an additional carbonyl group at C-11 (*δ*_C_ 202.3). The two methylene proton resonances at *δ*_H_ 2.68 And 2.49, which exhibit larger coupling constant (*J* = 16.4 Hz) and an HMBC correlation with *δ*_C_ 202.3 And 153.6 (Fig. 2), may be assigned to H_2_-12 in the *α*-position relative to one of the carbonyl groups (*δ*_C_ 202.3). Moreover, the large downfield chemical shift at C-8 (*δ*_C_ 150.3) and upfield chemical shift at C-9 (*δ*_C_ 153.6) also confirmed that the olefinic bond was assigned between C-7 carbonyl and C-11carbonyl in **4**. The relative configuration of compound **4** was determined by the following correlation signals on the NOESY spectrum: H_3_-19 and H_3_-28, H_3_-30; H_3_-30 and H-7; H-5 and H_3_-18, H_3_-29; H-20 and H_3_-18; H-24 and H_3_-27. The high match of the experimental and calculated ECD spectra showed that the absolute configurations of **4** were 5*R*,10*S*,13*S*,14*S*,17*S*,20*S* (Fig. 4)*.* Thus, the structure of **4** was confirmed as 3,7,11-trioxo-8,24*Z*-tirucalladien-26-oic acid.

Coggygrerol A (**5**) was a yellowish gum. The molecular formula of **5** was deduced from the quasi-molecular ion peak [M + Na]^+^ at *m/z* 463.3179 (calcd for C_29_H_44_O_3_Na, 463.3183) in the HRESIMS spectrum. The ^1^H and ^13^C NMR data (Table 2) indicated the presence of two ketone carbonyls [*δ*_C_ 202.1 (C-7), 205.1 (C-15)], two double bonds [*δ*_C_ 143.0 (C-8), 148.4 (C-14), 136.2 (C-22), 131.6 (C-23); *δ*_H_ 5.14 (1H, m, H-22), 5.14 (1H, m, H-23)], one oxygenated methine [*δ*_C_ 70.5 (C-3); *δ*_H_ 3.65 (1H, m, H-3)], and six methyl group [*δ*_C_ 18.2 (C-18), 12.5 (C-19), 21.7 (C-21), 12.4 (C-26), 21.2 (C-28), 19.3 (C-29); *δ*_H_ 1.01 (3H, s, H_3_-18), 0.95 (3H, s, H_3_-19), 1.10 (3H, d, *J* = 7.2 Hz, H_3_-21), 0.79 (3H, d, *J* = 6.9 Hz, H_3_-26), 0.83 (3H, d, *J* = 6.4 Hz, H_3_-28), 0.79 (3H, d, *J* = 6.4 Hz, H_3_-29)]. The ^1^H and ^13^C NMR spectra of compound **5** (Table 2) exhibited a high degree of similarity to those of the previously reported compound (3*S*,8*E*,22*E*)-hydroxystigmast-8,22-diene-7,11-dione [20] and as a typical steroidal skeleton. According to the subsequent comprehensive comparison of the data obtained for the two compounds, the bi-conjugate with a trans double bond (C-8, C-9) and two ketone carbonyl groups (C-7, C-11) in (3*S*,8*E*,22*E*)-hydroxystigmast-8,22-diene-7,11-dione was assumed to have transformed to ∆^8,14^ (double bond) and C-7/C-15 (two ketone carbonyl groups) in **5**. This assumption was confirmed by the HMBC correlations from H_2_-6 to C-7; H-9, H_2_-11 to C-8; H_2_-12, H-17 to C-14; and those from H_2_-16 to C-15 (Fig. 2). The configuration of the ∆^2,3^ double bond was deduced from the large coupling constant between the olefinic protons (*δ*_H_ 5.14, 1H, dd, *J* = 16.0, 8.2 Hz; *δ*_H_ 5.14, 1H, dd, *J* = 16.0, 8.2 Hz). The observed NOESY correlations between H-3/H-5, H-5/H-9, and H-9/H-17, determined that H-5, H-9, and H-17 were all located on the *α*-face of the steroid. Furthermore, the correlations between H_3_-18/H_3_-19 and H_3_-18/H-20 confirmed the *β*-orientation of C-18, C-19, and C-20 (Fig. 3). Based on the above evidence, the structure of **5** was unambiguously determined to be (3*S*,8*E*,22*E*)-hydroxystigmast-8,22-diene-7,15-dione.

**Table 2:** ^1^H NMR(400 MHz) and ^13^C NMR (100 MHz) data of **5** and **6** in CDCl_3_

No	**5**		**6**	
	*δ*_H_	*δ*_C_	*δ*_H_	*δ*_C_
1	1.79 (m)	36.1	1.78 (m)	36.1
	1.21 (m)		1.21 (m)	
2	1.90 (d, 13.0)	30.9	1.88 (m)	30.9
	1.44 (m)		1.42 (m)	
3	3.65 (m)	70.5	3.64 (m)	70.5
4	1.72 (m)	37.8	1.72 (m)	37.8
	1.42 (m)		1.42 (m)	
5	1.67 (m)	44.3	1.67 (m)	44.3
6	2.53 (d, 13.7)	46.5	2.53 (d, 14.1)	46.5
	2.35 (dd, 13.7, 3.3)		2.36 (dd, 14.1, 3.5)	
7		202.1		202.3
8		143.0		143.2
9	2.12 (m)	51.3	2.10 (m)	51.2
10		38.4		38.4
11	1.74 (m)	19.7	1.72 (m)	19.6
	1.57 (m)		1.56 (m)	
12	2.09 (m)	35.8	2.10 (m)	35.8
	1.34 (m)		1.29 (m)	
13		42.1		42.3
14		148.4		148.4
15		205.1		205.4
16	2.44 (dd, 19.6, 8.4)	41.6	2.53 (dd, 19.3, 8.4)	40.9
	2.00 (dd, 19.6, 11.8)		2.01 (dd, 19.3, 11.6)	
17	1.73 (m)	51.0	1.70 (m)	51.0
18	1.01 (s)	18.2	1.00 (s)	18.1
19	0.95 (s)	12.5	0.95 (s)	12.5
20	2.23 (dd, 16.3, 7.2)	40.0	1.57 (m)	35.3
21	1.10 (d, 7.2)	21.7	1.01 (d, 7.8)	19.2
22	5.14 (dd, 16.0, 8.2)	136.2	1.29 (m)	33.6
			1.12 (m)	
23	5.14 (dd, 16.0, 8.2)	131.6	1.15 (m)	25.7
24	1.55 (m)	51.4	0.91 (m)	45.8
25	1.42 (m)	25.3	1.46 (m)	29.1
	1.15 (m)			
26	0.79 (d, 6.9)	12.4	0.83 (d, 6.8)	12.1
27	1.54 (m)	31.9	1.22 (m)	23.2
28	0.83 (d, 6.4)	21.2	0.81 (d, 6.4)	19.0
29	0.79 (d, 6.4)	19.3	0.83 (d, 6.4)	19.9

Coggygrerol B (**6**) was a yellowish gum, and its HRESIMS showed *m/z* 465.3334 [M + Na]^+^, indicating a molecular formula of C_29_H_46_O_3_. By comparing the spectroscopic data (Table 2) of **6** with those of **5**, it was found that **6** had a structure similar to that of **5**, except that the double bond ∆^22,23^ was absent in **6**. The main difference in the ^1^H NMR spectrum was the absence of double bonds at *δ*_H_ 5.14 (2H), which was supported by the ^13^C NMR spectrum. These changes were verified via the ^1^H-^1^H COSY correlations (Fig. 2) of H-20/H_2_-22/H_2_-23/H-24, together with the HMBC relationships (Fig. 2) of H-20, H_3_-21 with C-22, and H-24, H_2_-25 with C-23. Thus, the structure of **6** was determined as (3*S*,8*E*)-hydroxystigmast-8-ene-7,15-dione.

By comparing spectroscopic data with those reported previously, the 27 known compounds isolated from this plant were shown to be aglaiabbreviatin E (**7**) [19], 3,7-dioxo-8,24*Z*-tirucalladien-26-oic acid (**8**) [21], 3-hydroxy-7-oxo-8,24*Z*-tirucalladien-26-oic acid (**9**) [21], 7,11-dioxo-3-hydroxy-8,24*Z*-tirucalladien-26-oic acid (**10**) [21], isomasticadienoic acid (**11**) [22], 24*Z*-isomasticadienolic acid (**12**) [23], (9*R*)-3,6-dioxo-tirucalla-7,24*Z*-dien-26-oic acid (**13**) [24], 3-hydroxy-6-oxo-7,24*Z*-tirucalladien-26-oic acid (**14**) [21], masticadienonic acid (**15**) [23], schinol (**16**) [21], 28-norolean-16,18-dien-3-one (**17**) [25], oleana-11:l3(18)-dien-3*β*-ol, (**18**) [26], *β*-amyrin (**19**) [27], cycloeucalenol (**20**) [28], obtusifolione (**21**) [29], obtusifoliol (**22**) [30], citrostadienol (**23**) [31], *β*-sitosterol (**24**) [32], 6-hydroxystigmast-4-en-3-one (**25**) [33], ergosta-4,6,8(14),22-tetraen-3-one (**26**) [34], butein (**27**) [35], sulfuretin (**28**) [36], 3,3',5',5,7-pentahydroflavanone (**29**) [37], fustin (**30**) [38], (−)-butin (**31**) [36], ( +)-butin (**32**) [35], And 5-hydroxyflavanone (**33**) [39].

### Effect of compounds on the LPS induced production of NO

**Fig. 5:**
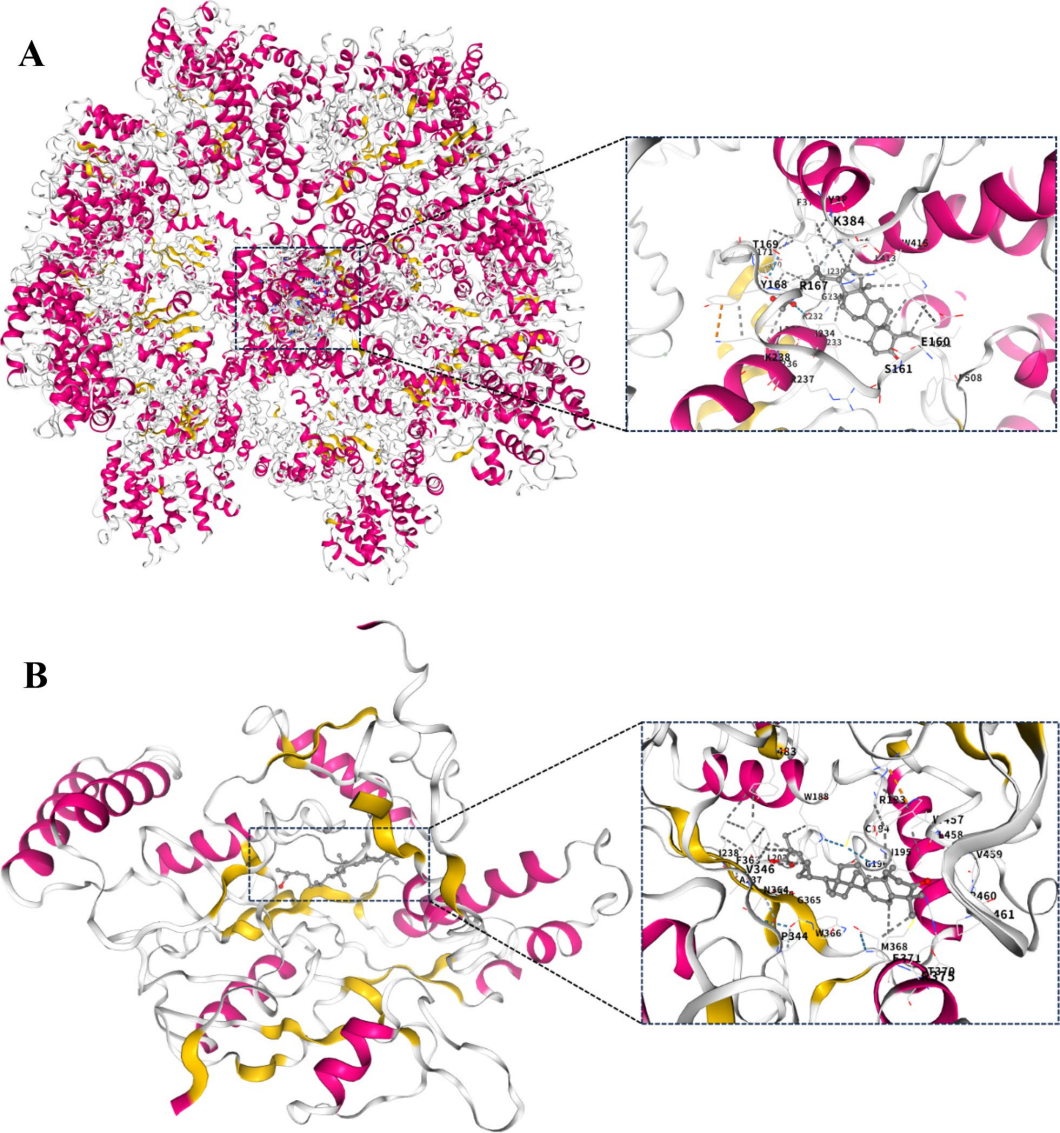
The molecular interactions of compound **15** with NLRP3 (A) and iNOS (B) by molecular docking simulation

Various diseases are accompanied by inflammation, and identifying small-molecule natural products with anti-inflammatory activity is a promising strategy for developing novel and effective therapeutic agents. Thus, the inhibitory effects of compounds **1**−**27** on LPS induced NO production in RAW 264.7 cells were assessed. Among them, compounds **5**, **13**, **15**, **21**, and **26** exhibited inhibitory activities in LPS induced RAW 264.7 cells, with IC_50_ values ranging from 6.81 ± 0.15 to 21.54 ± 0.35 μM (Table 3). Notably, compound **15** exhibited activity comparable to that of the positive control dexamethasone ( IC_50_:8.25 ± 0.6 μM). Molecular docking analysis revealed that compound **15** has a high affinity for NLRP3 and iNOS, with binding energies of −11.7 and −10.3 kcal/mol, respectively (Fig. 5). The above findings confirms that **15** exerts its anti-inflammatory effect by acting on NLRP3 and iNOS, revealing that NLRP3 and iNOS may be potential targets for the inhibitory activities of compound **15** in LPS-induced RAW 264.7 cells. These findings suggested that Coggygrerol A (**5**), (9*R*)-3,6-dioxo-tirucalla-7,24*Z*-dien-26-oic acid (**13**), masticadienonic acid (**15**), obtusifolione (**21**) and ergosta-4,6,8(14),22-tetraen-3-one (**26**) exhibited notable anti-inflammatory effects.

**Table 3:** Inhibitory effects of compounds on NO production induced by LPS in RAW 264.7 cells

Compound	IC_50_ (*μ*M)^a^
**5**	15.38 ± 0.20
**13**	11.26 ± 0.24
**15**	6.81 ± 0.15
**21**	21.54 ± 0.35
**26**	16.75 ± 0.44
Dexamethasone^b^	8.25 ± 0.6

^a^Half-inhibitory concentrations (IC_50_) in *μ*M

^b^Positive control

### Antibacterial activity of the isolated compounds in vitro

The inhibitory effects of the 27 triterpenoid And sterol compounds against the 10 bacterial strains were evaluated, and the results are presented in Table 4. Compounds **3**, **4**, **10**, **13**, **14**, and **26** exhibited varying inhibitory effects against the tested bacteria, while the other compounds showed no significant inhibitory effects. Notably, all six compounds contain multiple carbonyl groups and conjugated systems, which are thought to be related to their antibacterial activities. Specifically, compound **3** demonstrated noteworthy antibacterial activity against MRSA ATCC BAA-1717(USA300) And MRSA 12228, exhibiting a MIC value of 8 μg/mL.

**Table 4:** Antibacterial activity of compounds against test bacteria

**No**	MIC (*μ*g/mL)									
	G + bacteria					G- bacteria				
	*S.a.*^*a*^	*S.a.*^*b*^	*S.a.*^*c*^	*S.a.*^*d*^	*S.a.*^*e*^	*E.c.*^*f*^	*E.c.*^*g*^	*P.a.*^*h*^	*P.a.*^*i*^	*A.b.*^*j*^
**3**	16	8	8	16	32	128	64	64	> 128	64
**4**	16	16	32	16	64	> 128	128	64	128	64
**10**	128	64	64	128	128	> 128	> 128	> 128	64	> 128
**13**	32	32	128	64	64	64	128	128	> 128	64
**14**	64	64	> 128	> 128	128	> 128	> 128	128	64	> 128
**26**	64	16	16	32	64	32	32	64	128	32
ampicillin^k^	4	2	16	8	4	1	16	8	4	2
vancomycin^k^	1	0.5	1	0.5	1	8	4	4	2	8

^a^Methicillin-resistant *S. aureus* 171

^b^Methicillin-resistant *S. aureus* ATCC BAA-1717(USA300)

^c^Methicillin-resistant *S. aureus* ATCC 12228

^d^Methicillin-resistant *S. aureus* 209

^e^*S. aureus* ATCC 29213

^f^*E. coli* CMCC 44103

^g^*E. coli* CMCC 25922

^h^Multi-drug-resistant *P. aeruginosa* 110

^i^Multi-drug-resistant *P. aeruginosa* 174

^j^*A. baumannii* ATCC 19606

^k^Positive control

### Inhibitory effect of compound 3 on the growth of MRSA ATCC BAA-1717(USA300)

**Fig. 6:**
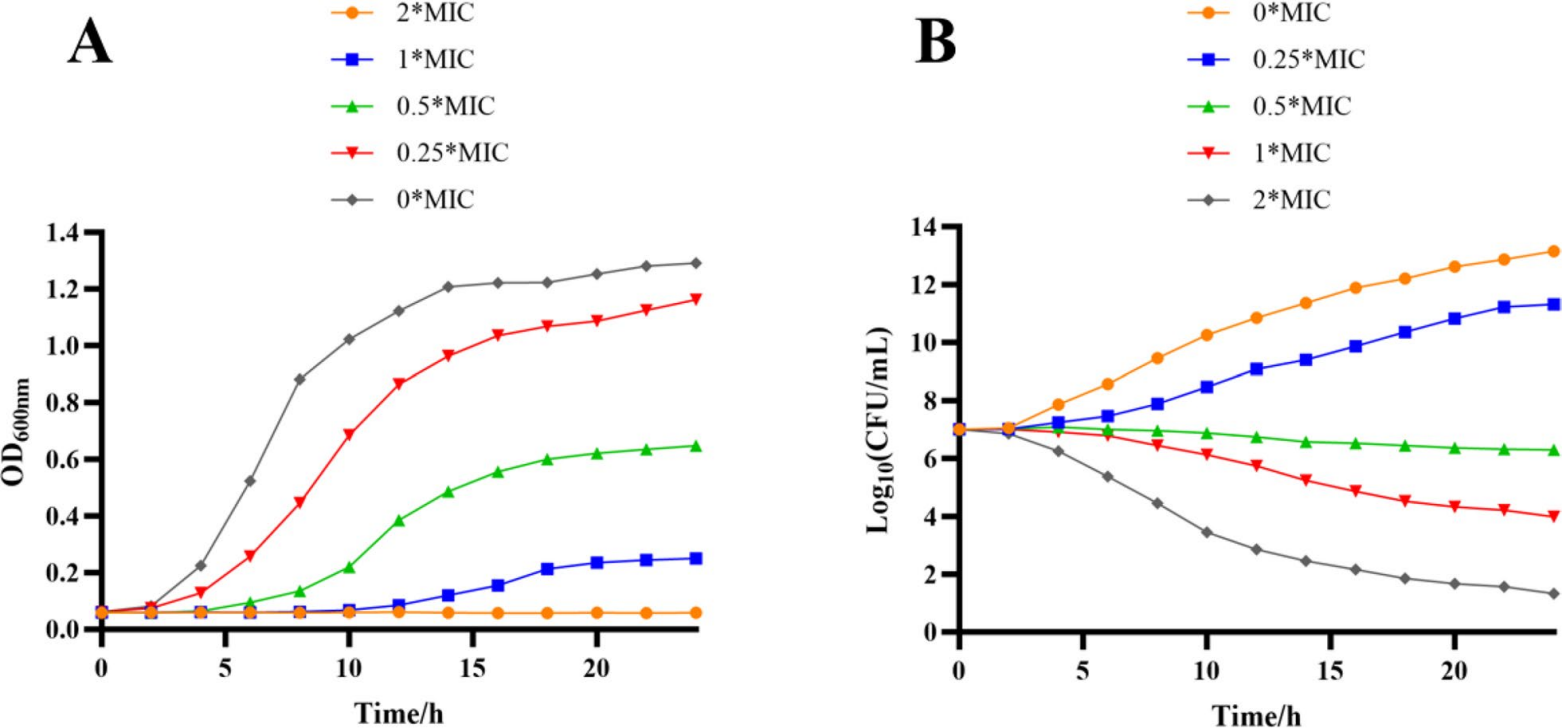
Growth curves (**A**) and Time-kill curves (**B**) of compound **3** against MRSA ATCC BAA-1717 (USA300)

The growth curves for MRSA ATCC BAA-1717(USA300) treated with compound **3** are shown in Fig. 6A. In the negative control group, the OD_600_ first increased rapidly And then remained stable until the end of the experiment. Compared to the control, 0.25 × MIC And 0.5 × MIC of **3** against MRSA ATCC BAA-1717(USA300) slightly delayed the exponential phase of the bacterial cells. The logarithmic period of the cells was significantly delayed when a higher concentration of compound **3** (1 × MIC) was used. In addition, at 2 × MIC of **3** against MRSA171, the growth curve remained almost horizontal from beginning to end (i.e., no logarithmic period occurred), suggesting that the growth of the cells was completely inhibited (Fig. 6A). Consequently, the growth curve analysis revealed that compound **3** exhibited potent dose- and time-dependent inhibitory effects against MRSA ATCC BAA-1717(USA300).

### Killing kinetics evaluation of compound 3 against MRSA ATCC BAA-1717(USA300)

In addition to the inhibition of bacterial growth, the rapid killing of bacteria is also a significant indicator of the antimicrobial activity of compounds. Consequently, compound **3** was selected for further evaluation of its bactericidal performance using a standard time-kill assay against MRSA ATCC BAA-1717(USA300). As illustrated in Fig. 6B, compound **3** rapidly eliminated MRSA ATCC BAA-1717(USA300) at 1 × MIC And 2× MIC.

### Extracellular alkaline phosphatase (AKP) activity

**Fig. 7:**
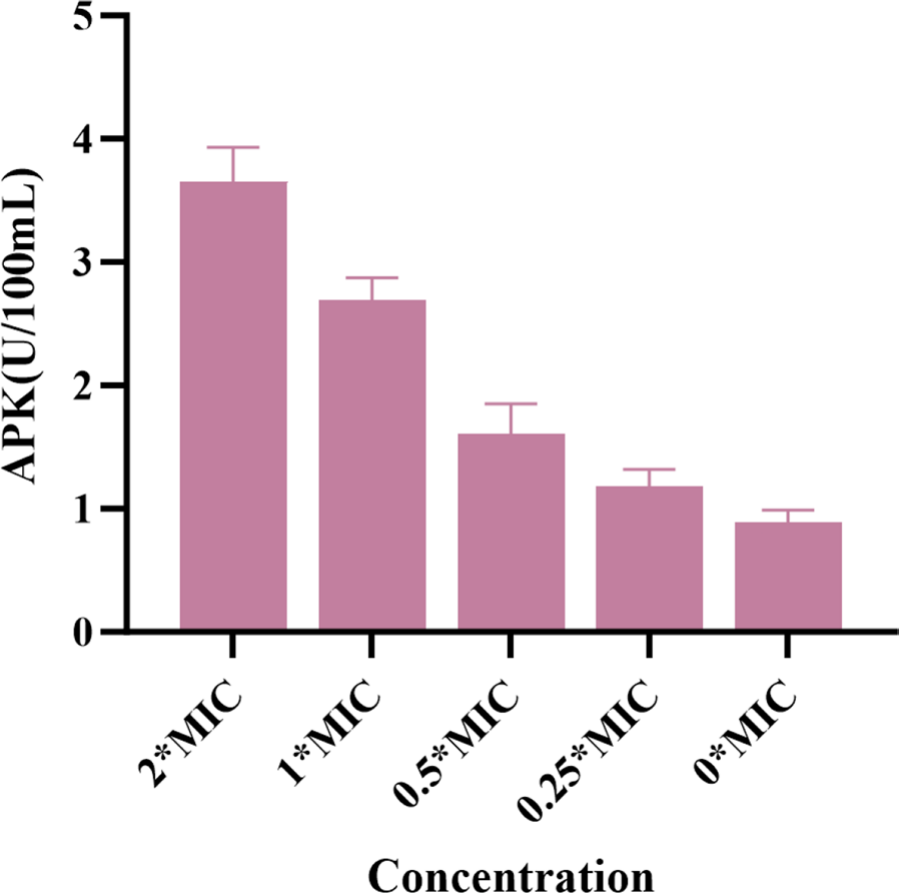
The effect of compound **3** on AKP activity in MRSA ATCC BAA-1717 (USA300)

The assessment of extracellular AKP levels can serve as a rapid indicator of cell wall damage and functional disruption. In this study, extracellular AKP levels were measured to evaluate the extent of cell wall damage caused by **3**. As shown in Fig. 7, the AKP level increased with the concentration of **3**. Compared to the blank control group, the AKP levels in the bacterial samples increased significantly. These findings suggest that **3** disrupts the cell wall, leading to AKP release.

### Antibiofilm activity

**Fig. 8:**
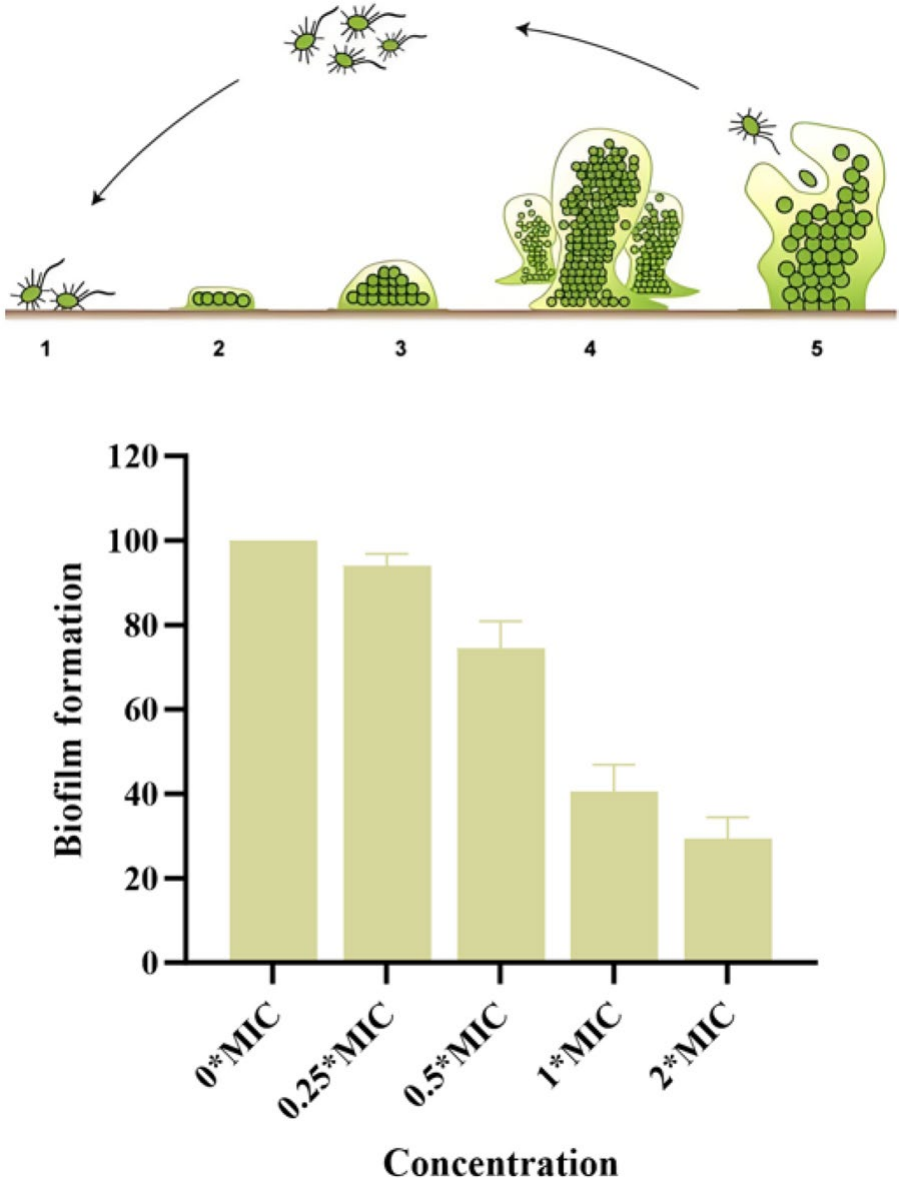
The effect of compound **3** on biofilm formation in MRSA ATCC BAA-1717 (USA300)

The effect of **3** on the biofilm formation capacity of MRSA ATCC BAA-1717(USA300) was evaluated using a crystal violet staining assay. Compound **3** exhibited a significant inhibitory effect on the biofilm formation of MRSA ATCC BAA-1717 (USA300). When treated with **3** at 2 × MIC, biofilm formation was inhibited by 70.5%. Treatment with **3** at 1 × and 1/2 × MIC inhibited biofilm formation by 59.4% And 25.5%, respectively (Fig. 8).

### SEM analysis of MRSA ATCC BAA-1717(USA300)

**Fig. 9:**
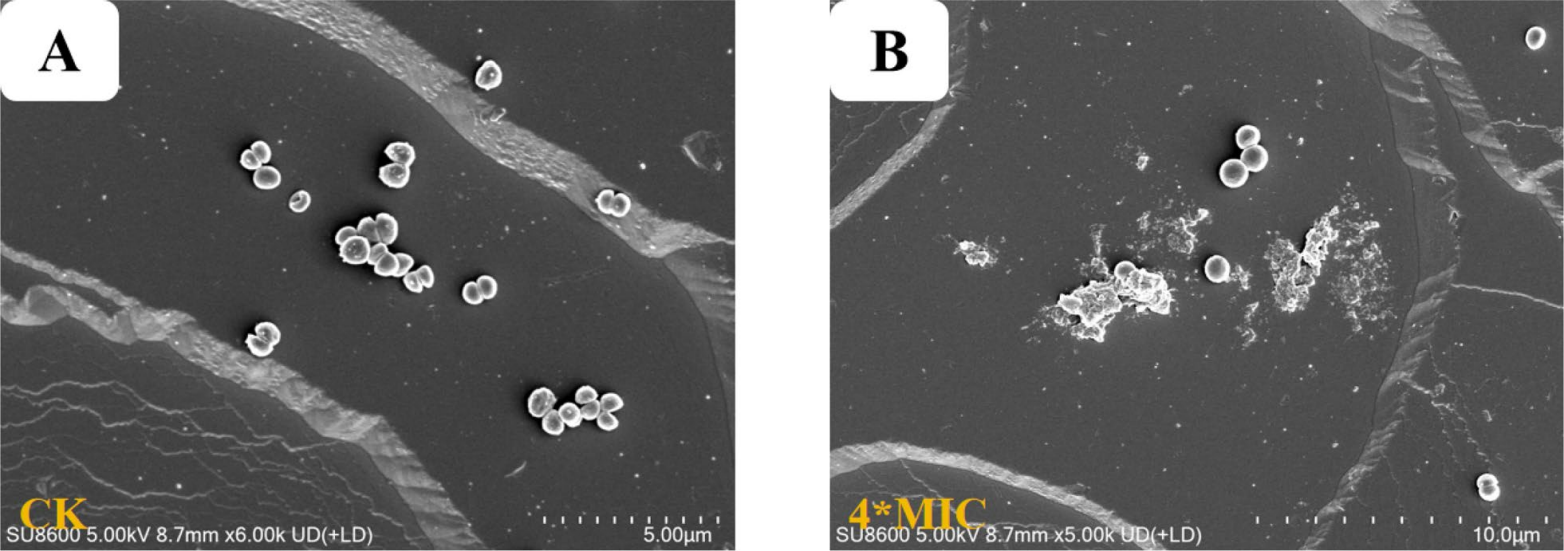
Scanning electron microscopy (SEM) images of MRSA ATCC BAA-1717 (USA300) treated with compound **3**. **A** Control for MRSA ATCC BAA-1717 (USA300). **B** Treated by compounds **3**

To further demonstrate the effects of **3** against MRSA ATCC BAA-1717(USA300), morphological alterations in the bacteria after treatment with **3** were examined using a Scanning Electron Microscope (SEM), and the resulting images are depicted in Fig. 9. Untreated MRSA ATCC BAA-1717(USA300) cells exhibited intact and smooth cell surfaces (Fig. 9A), whereas MRSA ATCC BAA-1717(USA300) cells treated with compound **3** at 4 × the MIC exhibited severe damage and breakage (Fig. 9B). According to these SEM results, it was inferred that the possible antibacterial mechanism of **3** against MRSA ATCC BAA-1717(USA300) might involve the induction of abnormal morphology in the cell membranes, causing an increase in membrane permeability.

### Combination susceptibility testing

**Fig. 10:**
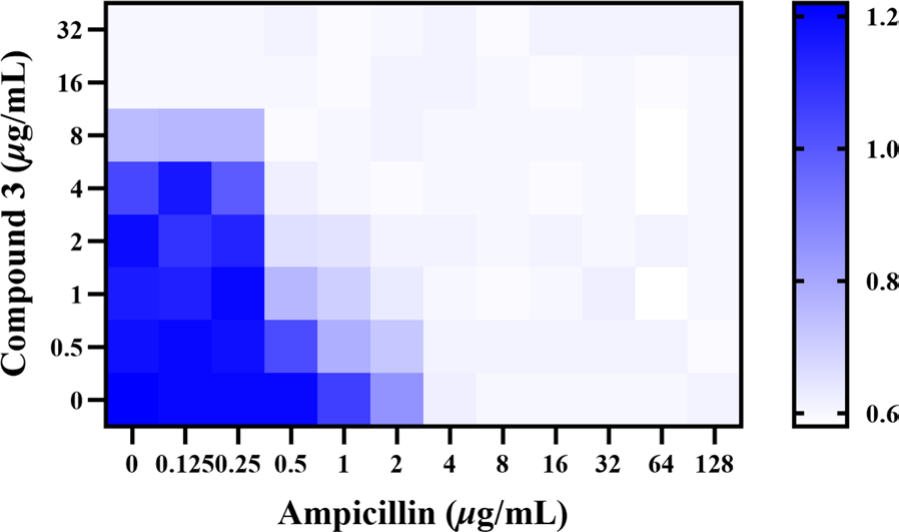
Antibacterial effect of the combination of compound **3** and ampicillin against MRSA ATCC BAA-1717 (USA300)

Combination susceptibility testing revealed that **3** and ampicillin exerted an additive effect against MRSA ATCC BAA-1717(USA300) (Fig. 10). This combination targets multiple bacterial sites, thereby reducing the likelihood of simultaneous resistance development to both mechanisms. Moreover, the use of this combination may enable dose reduction of each individual agent, potentially minimizing toxicity and delaying the emergence of resistance.

### Antibacterial activity of compound 3 in *vivo *in the *G. mellonella* infection model

**Fig. 11:**
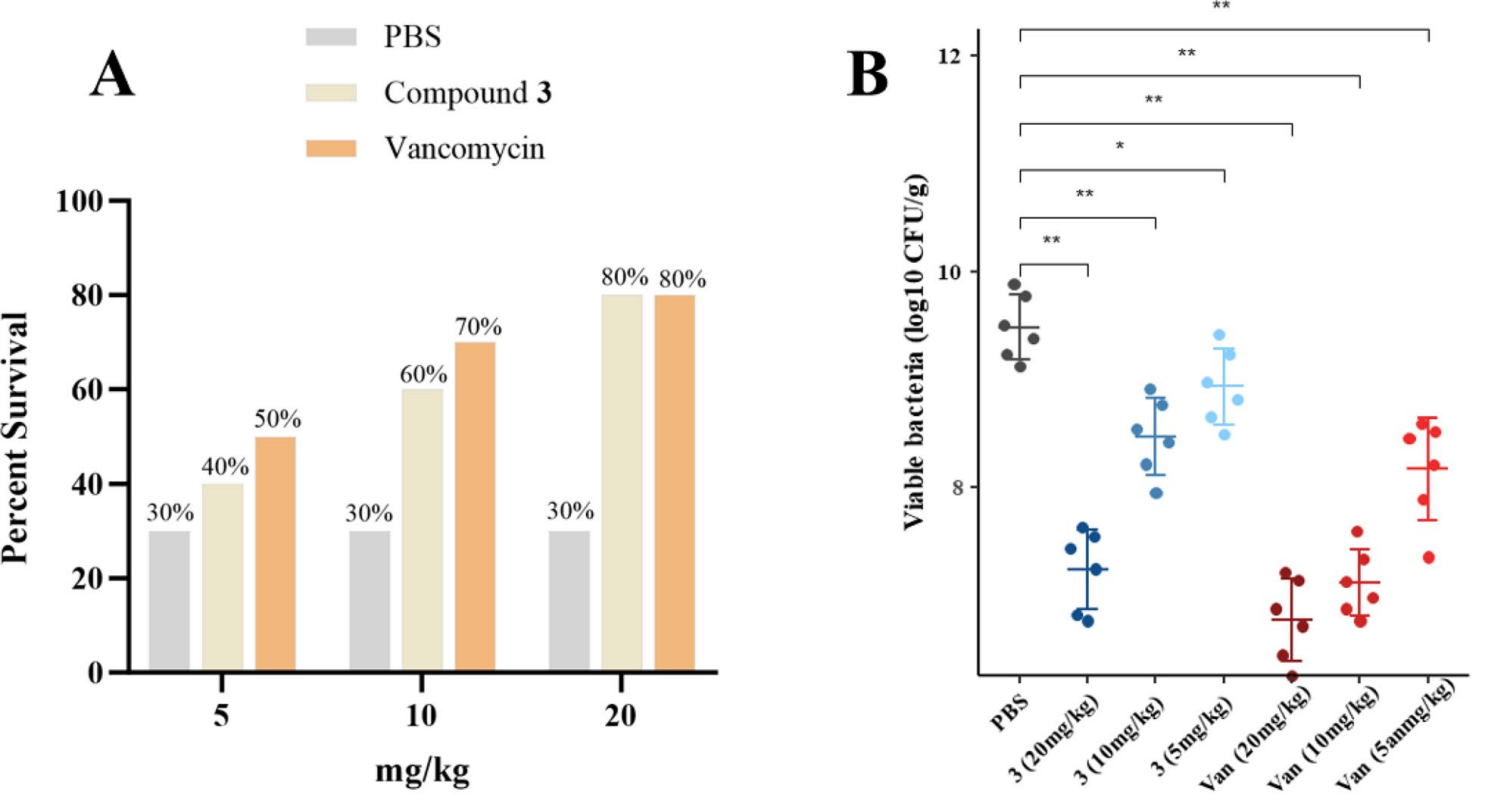
In *vivo* antibacterial activity of compound **3**. **A** Survival rate of infected *G. mellonella* after treatment with different concentrations of the drug (n = 10). **B** Bacterial content in *G. mellonella* (n = 6)

Based on the excellent antibacterial activity of compound **3**, it was further evaluated in *vivo* using a *G. mellonella* infection model with vancomycin as the positive control, as in the in *vitro* experiments. As shown in Fig. 11, treatment with **3** significantly increased the survival rate and reduced the bacterial content in the body in a dose-dependent Manner from 5 to 20 mg/kg. Moreover, at the high dose of 20 mg/kg, the treatment rate was 80%, consistent with that of the positive control group. These findings suggest that compound **3** has potential as a candidate for future antibiotic development.

## Conclusion

Six previously unreported compounds, triterpenoids (**1**−**4**) and sterols (**5** and **6**) And 27 known compounds (**7**−**33**) were isolated and identified from *C. coggygria*. Anti-inflammatory bioactivity evaluation showed that compounds **5**, **13**, **15**, **21**, and **26** significantly inhibited NO production induced by LPS in RAW264.7 cells, with IC_50_ values ranging from 6.81 ± 0.15 And 21.54 ± 0.35 μM. The molecular docking results indicated the potential of NLRP3 and iNOS as target proteins for compound **15**. Furthermore, the isolated triterpenoids and sterols were evaluated for their antibacterial activity against ten bacterial strains, revealing that compound **3** was the most active, with significant inhibitory activity against MRSA ATCC BAA-1717 (USA300). Subsequent analysis of the antibacterial mechanism indicated that compound **3** could potentially lead to the destruction of bacterial cell walls and membranes. Additionally, a synergistic effect was observed when **3** was combined with ampicillin. Compound **3** also exhibited antibacterial activity in a *G. mellonella* infection model. These findings provide a preliminary molecular and biological basis for the anti-inflammatory and antibacterial activities of small-molecule components in *C. coggygria*. In addition to being an ornamental tree species, *C. coggygria* may be developed as a value-added medicinal plant because of its highlighted antibacterial potential.

## Experimental

### General experimental procedures

The general experimental procedures were carried out in accordance with a previous report and are detailed in Text S1 [40].

### Plant material

The tree stems and bark of *C. coggygria* were collected in October 2023 from the Tsinling Mountains of China. The plant species were identified by Dr. Zhen-Hai Wu, College of Life Sciences, Northwest A&F University. A voucher specimen (no. WUK 0480712) was deposited in the Herbarium of the College of Life Sciences, Northwest A&F University.

### Extraction and isolation

The dried and crushed tree stems and bark of *C. coggygria* (30.0 kg) were subjected to three rounds of extraction with eight times their volume of heated 95% ethanol, resulting in a 4.5 kg crude extract. Subsequently, the crude extract was dissolved in three times the volume of water and partitioned sequentially with equal volumes of petroleum ether, and EtOAc, resulting in three partitions. Silica gel flash column chromatography (FCC) with dichloromethane/MeOH (80:1 to 2:1) was used to purify EtOAc fraction C (600.0 g), resulting in four fractions (A−D). Fraction A (30.0 g) was further purified using an FCC silica gel column and eluted with PE/EtOAc (40:1 to 0:1), resulting in five subfractions (A1−A5). Hereafter, A2 (1.4 g) was divided into four subfractions (A2-1−A2-4) using FCC silica gel with petroleum ether/EtOAc (20:1 to 2:1). The A2-1 (203.0 mg) and A2-2 (120.0 mg) fractions were separated by semipreparative HPLC to obtain compounds **3** (MeOH/H_2_O, 95:5; flow rate: 2 mL/min; 3.5 mg; *t*_R_ = 22.2 min), **7** (MeOH/H_2_O, 95:5; flow rate: 2 mL/min; 8.8 mg; *t*_R_ = 29.1 min), **16** (MeOH/H_2_O, 98:2; flow rate: 2 mL/min; 7.6 mg; *t*_R_ = 18.2 min), and **17** (MeOH/H_2_O, 98:2; flow rate: 2 mL/min; 18.0 mg; *t*_R_ = 25.0 min), respectively. Futher, Fraction A2-3 (60.0 g) was separated on a silica gel column eluted with PE/EtOAc (5:1) to give compounds **11** (10.5 mg) and **12** (7.6 mg). Fraction A3 (5.3 g) was separated on a silica gel column using PE/EtOAc (20:1 to 2:1) elution to yield subfractions A3-1 and A3-2. Fractions A3-1 (1.2 g) and A3-2 (1.9 g) were separated by chromatography on a Sephadex LH-20 column and eluted with dichloromethane/MeOH (1:1) and then purified using semi-preparative HPLC, which yielded compounds **4** (MeOH/H_2_O, 90:10; flow rate: 2 mL/min; 10.8 mg; *t*_R_ = 20.1 min), **18** (MeOH/H_2_O, 90:10; flow rate: 2 mL/min; 12.0 mg; *t*_R_ = 23.8 min), **19** (MeOH/H_2_O, 92:8; flow rate: 2 mL/min; 35.0 mg; *t*_R_ = 23.1 min), **24** (MeOH/H_2_O, 92:8; flow rate: 2 mL/min; 26.0 mg; *t*_R_ = 28.9 min), and **25** (MeOH/H_2_O, 92:8; flow rate: 2 mL/min; 8.0 mg; *t*_R_ = 32.0 min). Fraction A4 (3.1 g) was separated on a silica gel column eluted with PE/EtOAc (20:1 to 1:1) to give fractions A4-1−A4-5. A4-1 (188.0 mg) was further separated on silica gel CC (PE/EtOAc, 12:1) followed by elution to yield **8** (18.4 mg), **9** (22.0 mg), and **15** (10.8 mg). Fraction A4-2 (550.0 mg), on the other hand, was subjected to chromatography on Sephadex LH-20 (Me_2_CO) and then purified using HPLC (MeOH/H_2_O, 89:11), which yielded compound **1** (3.3 mg, *t*_R_ = 45.8 min) and **2** (2.8 mg, *t*_R_ = 47.2 min). A4-3 (1.1 g) was applied to silica gel CC eluted with a gradient of PE/EtOAc (20:1 to 5:1), to give five subfractions (A4-3–1−A4-3–4). Section A4-3–2 (65.0 mg) was purified by semipreparative HPLC (MeOH/H_2_O, 85:15; flow rate: 2 mL/min) to yield compounds **5** (6.0 mg; *t*_R_ = 26.6 min) and **6** (6.5 mg; *t*_R_ = 28.0 min). Similarly, compounds **10** (4.5 mg; *t*_R_ = 32.0 min) and **13** (9.8 mg; *t*_R_ = 35.2 min) were isolated from subfraction A4-3–3 (320.0 mg) using semi-preparative HPLC (MeOH/H_2_O, 86:14; flow rate: 2 mL/min). As well, A4-3–4 (980.0 mg) was subjected to Sephadex LH-20 column elution with MeOH to obtain subfractions A4-3-4-1 and A4-3-4-2. Compound **14** (26.0 mg; *t*_R_ = 24.3 min) was isolated from subfraction A4-3-4-1 (310.0 mg) by semipreparative HPLC (MeOH/H_2_O, 81:19; flow rate: 2 mL/min). Additionally, compounds **20** (4.5 mg; *t*_R_ = 20.8 min), **21** (5.0 mg; *t*_R_ = 34.5 min), and **26** (85.0 mg; *t*_R_ = 38.0 min) were isolated from A4-3-4-2 (525.0 mg) by semipreparative HPLC (MeOH/H_2_O, 84:16; flow rate: 2 mL/min). Fraction B (15.0 g) was isolated using silica gel CC (PE/EtOAc, 20:1 to 1:1) to obtain three fractions (B1−B3). B1 (1.5 g) was then purified over a Sephadex LH-20 column and eluted with dichloromethane/MeOH to afford B1-1− B1-4. Separation of B1-2 (160.0 mg) on semipreparative HPLC (MeOH/H_2_O, 88:12; flow rate: 2 mL/min) afforded compounds **22** (12.8 mg; *t*_R_ = 20.6 min) and **23** (3.8 mg; *t*_R_ = 23.9 min). Fraction C (300.0 g) was separated on a silica gel column chromatograph using gradient elution (dichloromethane/MeOH, 40:1 to 5:1), yielding five subfractions (C1−C5). Fraction C2 (16.0 g) was further separated on a silica gel column and eluted with dichloromethane/MeOH (40:1 to 10:1) to obtain fractions C2-1−C2-6. Purification of fraction C2-2 (1.3 g) via semipreparative HPLC (MeOH/H_2_O, 74:26; flow rate: 2 mL/min) yielded compounds **27** (7.5 mg; *t*_R_ = 19.2 min), **28** (4.6 mg; *t*_R_ = 25.0 min), **31** (75.0 mg; *t*_R_ = 27.6 min), **32** (86.0 mg; *t*_R_ = 29.4 min), and **33** (24.5 mg; *t*_R_ = 35.2 min). Fraction C2-3 (2.1 g) was purified by semi-preparative HPLC (MeOH/H_2_O, 76:24; flow rate: 2 mL/min) to yield compound **29** (19.0 mg, *t*_R_ = 20.0 min)and **30** (46.0 mg; *t*_R_ = 26.9 min).

Coggygrnoid A (**1**): Colorless solid; $[\alpha]^{\text D}_{20}$+ 45.0 (*c* 0.1, MeOH); UV (MeOH) *λ*max (log *ε*): 228.0 (2.55) nm; CD (MeOH) *λ*_max_ (*Δε*) 210 (– 5.79), 233 (+ 1.05); ^1^H and ^13^C NMR data see Table 1; HRESIMS [M + Na]^+^ *m*/*z* 461.3382 (calcd for C_30_H_46_O_2_Na, 461.3390).

Coggygrnoid B (**2**): Colorless solid; $[\alpha]^{\text D}_{20}$ + 60.0 (*c* 0.1, MeOH); UV (MeOH) *λ*_max_ (log *ε*): 226.0 (2.29) nm; ^1^H and ^13^C NMR data see Table 1; HRESIMS [M + Na]^+^ *m*/*z* 461.3395 (calcd for C_30_H_46_O_2_Na, 461.3390).

Coggygrnoid C (**3**): Faint yellowish gum; $[\alpha]^{\text D}_{20}$ + 14.0 (*c* 0.1, MeOH); UV (MeOH) *λ*_max_ (log *ε*): 281.0 (2.30) nm; ^1^H and ^13^C NMR data see Table 1; HRESIMS [M + Na]^+^ *m*/*z* 477.3330 (calcd for C_30_H_46_O_3_Na, 477.3339).

Coggygrnoid D (**4**): Yellowish gum; $[\alpha]^{\text D}_{20}$ − 28.0 (*c* 0.1, MeOH); UV (MeOH) *λ*_max_ (log *ε*): 269.0 (2.13) nm; CD (MeOH) *λ*_max_ (*Δε*) 203 (– 9.99), 231 (– 10.71), 273 (+ 11.25); ^1^H and ^13^C NMR data see Table 1; HRESIMS [M−H]^−^ *m/z* 481.2957 (calcd for C_30_H_41_O_5_, 481.2959).

Coggygrerol A (**5**): Yellowish gum; $[\alpha]^{\text D}_{20}$ + 26.0 (*c* 0.1, MeOH); UV (MeOH) *λ*_max_ (log *ε*): 200.0 (2.52) nm; CD (MeOH) *λ*_max_ (*Δε*) 201 (+ 8.91), 218 (+ 8.58); ^1^H and ^13^C NMR data see Table 2; HRESIMS [M + Na]^+^ *m/z* 463.3179 (calcd for C_29_H_44_O_3_Na, 463.3183).

Coggygrerol B (**6**): Yellowish gum; $[\alpha]^{\text D}_{20}$+ 24.0 (*c* 0.1, MeOH); UV (MeOH) *λ*_max_ (log *ε*): 200.0 (2.34), 258.0 (1.98) nm; CD (MeOH) *λ*_max_ (*Δε*) 206 (+ 11.23), 319 (– 2.03), 273 (+ 11.25); ^1^H and ^13^C NMR data see Table 2; HRESIMS [M + Na]^+^ *m/z* 465.3334 (calcd for C_29_H_46_O_3_Na, 465.3339).

### Anti-inflammatory assay

As outlined in our previous article, the MTT assay and Griess reaction were employed to measure cell viability and NO production [41]. Futhermore, Molecular docking studies was operated as described previously [41].

### Anti-bacterial assay

The effects of compounds **1**−**27** against the five gram-positive bacteria (Methicillin-resistant *S. aureus* 171, Methicillin-resistant *S. aureus* ATCC BAA-1717 (USA300), Methicillin-resistant *S. aureus* ATCC 12228, Methicillin-resistant *S. aureus* 209, and *S. aureus* ATCC 29213) and five gram-negative bacteria (*E. coli* CMCC 44103, *E. coli* CMCC 25922, Multi-drug-resistant *P. aeruginosa* 110, Multi-drug-resistant *P. aeruginosa* 174, and *A. baumannii* ATCC 19606) were evaluated in *vitro*. The minimal inhibitory concentration (MIC) was determined for compounds **1**–**27** using the antibacterial activity test protocols described in previous reports [42]. Based on previously reported methods [42], the growth curve of MRSA171 treated with compound **3** were evaluated. The previously described method was used to determine the time-kill curve of the test bacteria [43].

### Determination of alkaline phosphatase (AKP) activity

Following an 8-h co-incubation of MRSA ATCC BAA-1717 (USA300) with **3**, culture supernatants from each treatment group were collected. The concentration of alkaline phosphatase (AKP) was subsequently determined using a commercial AKP assay kit, following the manufacturer's protocol [44].

### Assay for biofilm suppression

MRSA ATCC BAA-1717 (USA300) was incubated with **3** for 24 h. After incubation, the medium was removed And the biofilms were washed twice with PBS. Then, 0.1% crystal violet solution was added to each well And incubated for 30 min. The dye was dissolved by adding 33% glacial acetic acid after staining. After incubating for 1 h, the absorbance at OD_590_ was determined using a microplate reader [45].

### Scanning electron microscopy (SEM)

According to our method, with minor modifications [42], a scanning electron microscopy (SEM) study was conducted to observe the cellular morphological changes in MRSA ATCC BAA-1717 (USA300) treated with **3**. MRSA ATCC BAA-1717 (USA300) (approximately 2 × 10^8^ CFU/mL) was treated with compound **3** at 4 × MIC. After culturing for 10 h at 37 °C, MRSA ATCC BAA-1717 (USA300) was collected, washed with PBS (0.1 M, pH7.0) and then anchored with glutaraldehyde. Field-emission scanning electron microscopy (FE-SEM; Nova Nano SEM-450, FEI Instruments, Inc. Hillsboro, Oregon) was used for the microstructural evaluation of MRSA ATCC BAA-1717 (USA300).

### Checkerboard MIC assay

The microdilution checkerboard method was used to evaluate the antibacterial effect of compound **3** in combination with ampicillin against MRSA ATCC BAA-1717 (USA300). Specifically, two-fold serial dilutions of **3** And ampicillin were prepared in a 96-well microtiter plate And inoculated with a bacterial suspension at a final concentration of 1× 10^8^ CFU/mL. After incubation at 37 °C for 24 h, the interaction between the two agents was assessed using the fractional inhibitory concentration index (FICI) formula [46].

$${\mathrm{FICI}}\, = \,\left( {{\mathrm{MIC}}_{{\text{A in combination}}} /{\mathrm{MIC}}_{{\text{A alone}}} } \right)\, + \,({\mathrm{MIC}}_{{\text{B in combination}}} /{\mathrm{MIC}}_{{\text{B alone}}} )$$

### *G. mellonella* infection model and in vivo antibiotic activity assessment

*G. mellonella* larvae are used widely as surrogate infectious diseasemodels,due to ease ofuse and the presence of an innate immune system functionally similar to that of vertebrates [47]. To Analyze the survival of infected larvae, 5 *μ*L of the bacterial inoculum (1 × 10^8^ CFU/mL) was injected dorsolaterally into the hemocoel of the last-instar larvae. Following An incubation period at 37 °C for 60 min And observation that the larvae continued to survive normally, 5 *μ*L of each working solution of compound **3** or the positive drugs (5, 10, And 20 mg/kg) were injected. The larvae were deemed deceased when they exhibited An absence of tactile response following a 72-h incubation at 37 °C. Similarly, the second batch of *G. mellonella* was inoculated with bacteria And treated with different concentrations of the drug using the same method as above. After 24 h, the entire *G. mellonella* homogenate was suspended in PBS, then serially diluted tenfold to 10^8^-fold. A 100 *μ*L aliquot was spread onto agar plates, And colony counts were performed after 18 h (expressed in log^10^ CFU/g).

### Statistical analysis

All of the biological experimental data presented in this paper were replicated three times, and the results are shown as the mean ± standard deviation (SD).

## Supplementary Information

Additional file 1.

## Data Availability

Upon reasonable request, data utilised or analysed during this study may be obtained from the corresponding author.
