# Supplementary material for: First discovery of triterpenoids and sterols from Cotinus coggygria var. cinereus Engl. with anti-inflammatory and antibacterial activities
Source: Nat Prod Bioprospect. 2026 Jan 5;16(1):1. doi: 10.1007/s13659-025-00553-4 (PMC12765759; doi:10.1007/s13659-025-00553-4)
Supplement: Supplementary file 1 — Additional file 1. [file 13659_2025_553_MOESM1_ESM.docx]

**Supporting Information**

**First discovery of triterpenoids and sterols from *Cotinus coggygria* var. cinereus Engl. with anti-inflammatory and antibacterial activities**

Yue-Tong Zhu^1^, Ze-Rui Li^1^, Ren-Hao Chen^1^, Jin-Hao Li^1^, Wei-Wang^1^, Yu-Qi Gao*^,2^, Chun-Huan Li*^,1^, Jin-Ming Gao*^,1^

^1^Shaanxi Key Laboratory of Natural Products & Chemical Biology, College of Chemistry & Pharmacy, Northwest A&F University, 712100 Yangling, People’s Republic of China.

^2^College of Food Science and Technology, Northwest University, Xi'an 710069, Shaanxi, People's Republic of China.

Corresponding Authors: [gyq1225@nwu.edu.cn](mailto:gyq1225@nwu.edu.cn) (YQ Gao); chunhuanli@nwsuaf.edu.cn (CH Li); jinminggao@nwsuaf.edu.cn (JM Gao)

**List of supporting information**

Text S1. General experimental procedures

Text S2. ECD Calculations

Figure S1. ^1^H NMR spectrum of compound **1** (400MHz, CDCl_3_)

Figure S2. ^13^C NMR spectrum of compound **1** (100MHz, CDCl_3_)

Figure S3. ^1^H-^1^H COSY spectrum of compound **1**

Figure S4. HSQC spectrum of compound **1**

Figure S5. HMBC spectrum of compound **1**

Figure S6. NOESY spectrum of compound **1**

Figure S7. HR-ESI-MS spectrum of compound **1**

Figure S8. UV spectrum of compound **1** (MeOH)

Figure S9. ^1^H NMR spectrum of compound **2** (400MHz, CDCl_3_)

Figure S10. ^13^C NMR spectrum of compound **2** (100MHz, CDCl_3_)

Figure S11. ^1^H-^1^H COSY spectrum of compound **2**

Figure S12. HSQC spectrum of compound **2**

Figure S13. HMBC spectrum of compound **2**

Figure S14. NOESY spectrum of compound **2**

Figure S15. HR-ESI-MS spectrum of compound **2**

Figure S16. UV spectrum of compound **2** (MeOH)

Figure S17. ^1^H NMR spectrum of compound **3** (400MHz, CDCl_3_)

Figure S18. ^13^C NMR spectrum of compound **3** (100MHz, CDCl_3_)

Figure S19. ^1^H-^1^H COSY spectrum of compound **3**

Figure S20. HSQC spectrum of compound **3**

Figure S21. HMBC spectrum of compound **3**

Figure S22. NOESY spectrum of compound **3**

Figure S23. HR-ESI-MS spectrum of compound **3**

Figure S24. UV spectrum of compound **3** (MeOH)

Figure S25. ^1^H NMR spectrum of compound **4** (400MHz, CDCl_3_)

Figure S26. ^13^C NMR spectrum of compound **4** (100MHz, CDCl_3_)

Figure S27. ^1^H-^1^H COSY spectrum of compound **4**

Figure S28. HSQC spectrum of compound **4**

Figure S29. HMBC spectrum of compound **4**

Figure S30. NOESY spectrum of compound **4**

Figure S31. HR-ESI-MS spectrum of compound **4**

Figure S32. UV spectrum of compound **4** (MeOH)

Figure S33. ^1^H NMR spectrum of compound **5** (400MHz, CDCl_3_)

Figure S34. ^13^C NMR spectrum of compound **5** (100MHz, CDCl_3_)

Figure S35. ^1^H-^1^H COSY spectrum of compound **5**

Figure S36. HSQC spectrum of compound **5**

Figure S37. HMBC spectrum of compound **5**

Figure S38. NOESY spectrum of compound **5**

Figure S39. HR-ESI-MS spectrum of compound **5**

Figure S40. UV spectrum of compound **5** (MeOH)

Figure S41. ^1^H NMR spectrum of compound **6** (400MHz, CDCl_3_)

Figure S42. ^13^C NMR spectrum of compound **6** (100MHz, CDCl_3_)

Figure S43. ^1^H-^1^H COSY spectrum of compound **6**

Figure S44. HSQC spectrum of compound **6**

Figure S45. HMBC spectrum of compound **6**

Figure S46. NOESY spectrum of compound **6**

Figure S47. HR-ESI-MS spectrum of compound **6**

Figure S48. UV spectrum of compound **6** (MeOH)

Text S1. General experimental procedures

Ultraviolet (UV) and circular dichroism (CD) spectra were obtained using an Applied Photophysics Chirascan spectrometer (Applied Photophysics, Ltd., Surrey, UK). Optical rotations were determined using an Autopol III polarimeter (Rudolph Research Analytical). Nuclear magnetic resonance (NMR) spectra were acquired using two Bruker Avance spectrometers, Neo 400 and III-500, with tetramethylsilane (TMS) as the internal standard. HRESIMS data were obtained using an LC-30A + TripleTOF5600 + system (AB SCIEX, Singapore). Sephadex LH-20 (GE Healthcare, Boston, MA, USA), and silica gel (100~200 mesh, 200~300 mesh, Qingdao Marine Chemical Co., Ltd., Qingdao, China) were employed for column chromatography (CC). Semi-preparative HPLC was conducted on an Agilent 1100 series system utilizing a YMC C18 column (250 mm × 10 mm, i.d., 5 *μ*m). Thin-layer chromatography (TLC) was performed on silica gel 60F254 and RP-18 F254S plates (Merck KGaA, Darmstadt, Germany). All other chemicals used in this study were of analytical grade. Scanning Electron Microscopy (SEM; Hitachi, S-3400N, Japan) was used to analyze the aggregation status and morphology.

Text S2. ECD Calculations

Gaussian 16 software was used to perform ECD calculations. Conformation optimization was carried out in the gas phase using density functional theory (DFT) at the B3LYP/6-31G(d) level. Time-dependent density functional theory was also employed. (TDDFT) ECD calculations were carried out in MeOH (PCM) using the B3LYP/6-311G(d,p) level, and ECD spectra were obtained with SpecDis 1.7.


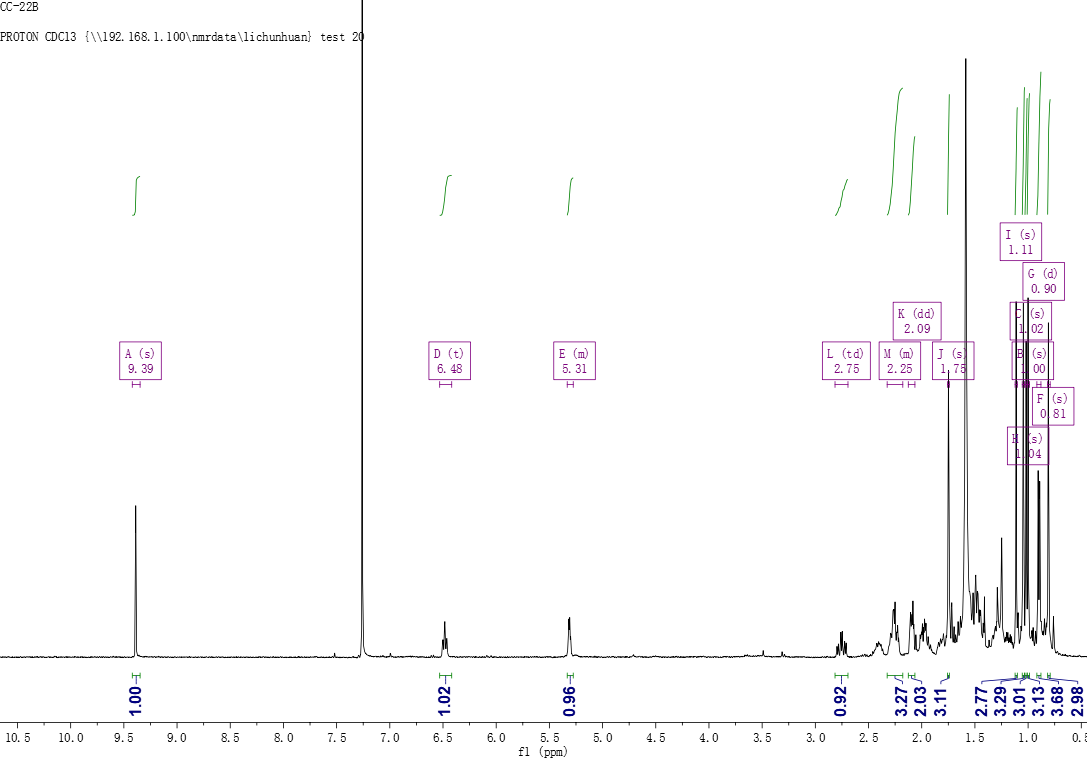


Figure S1. ^1^H NMR spectrum of compound **1** (400MHz, CDCl_3_)


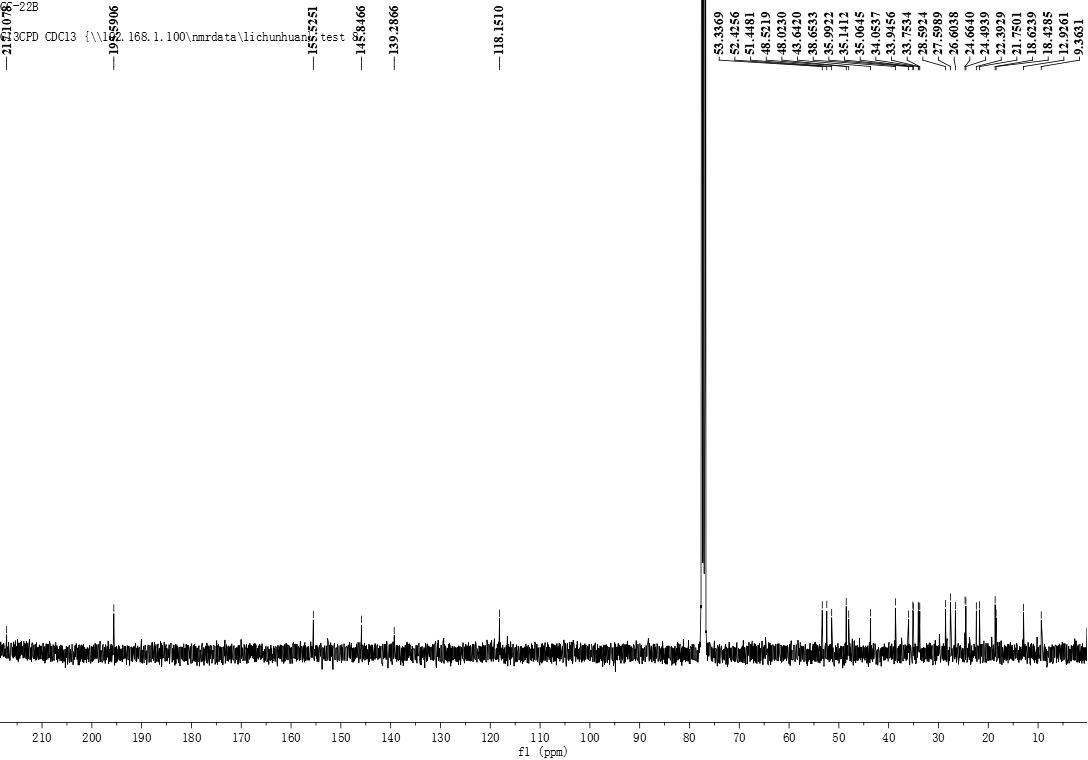


Figure S2. ^13^C NMR spectrum of compound **1** (100MHz, CDCl_3_)


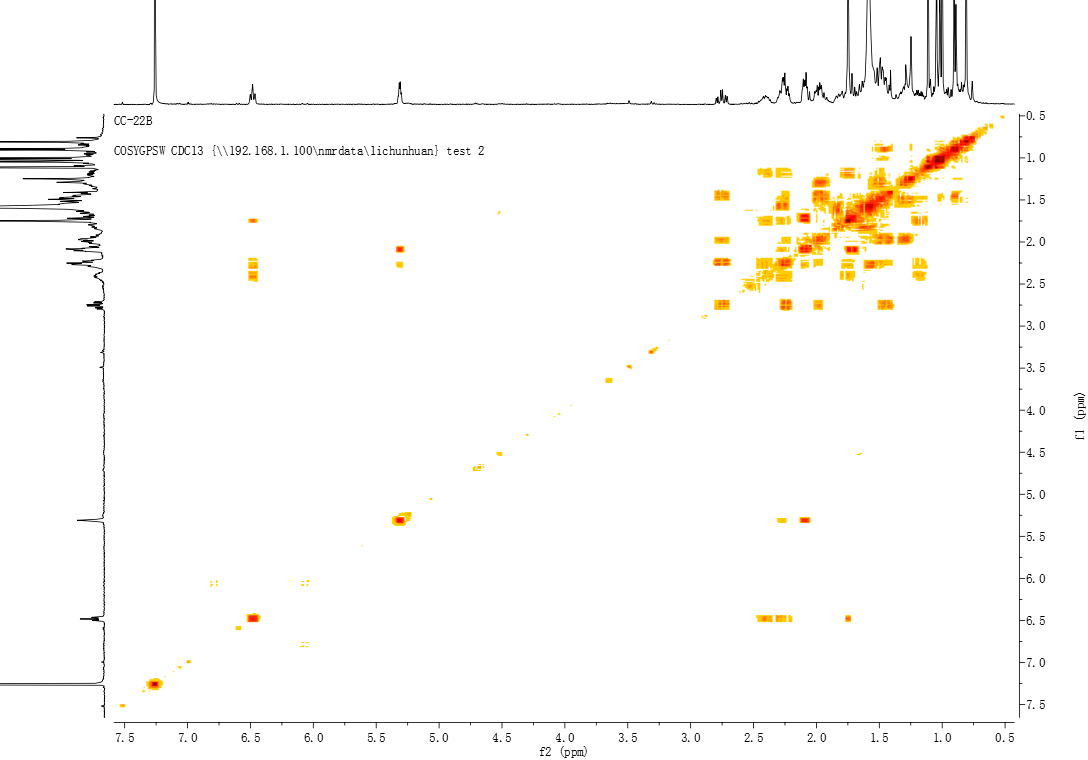


Figure S3. ^1^H-^1^H COSY spectrum of compound **1**


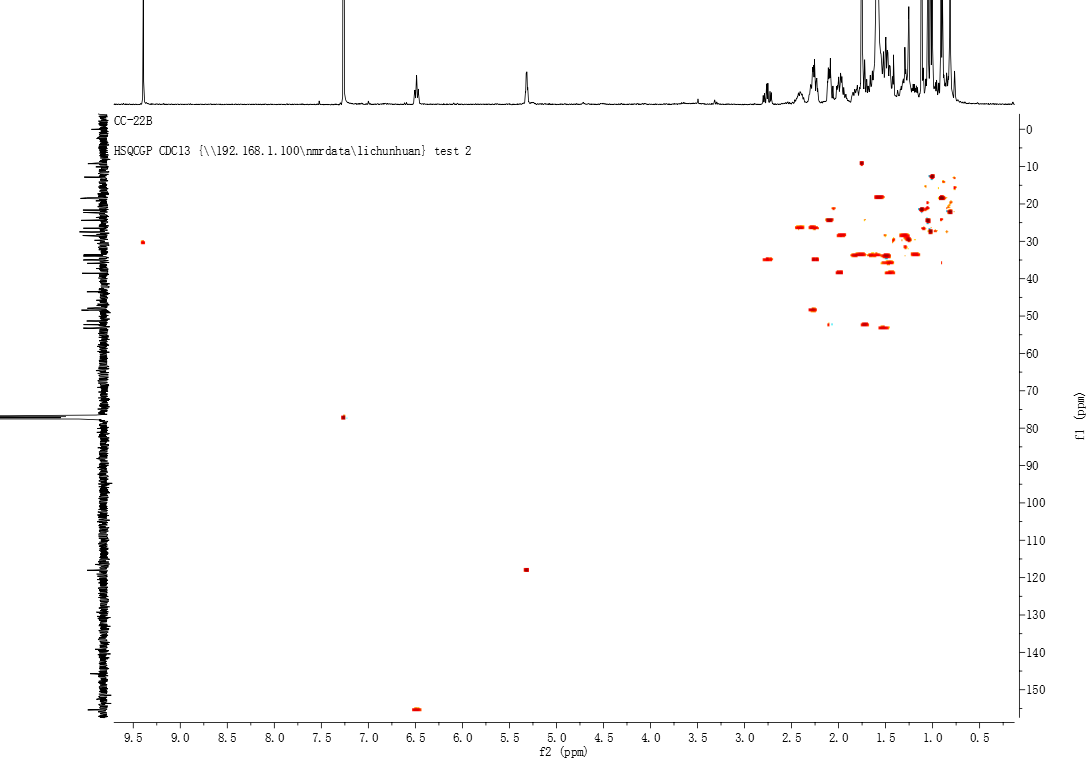


Figure S4. HSQC spectrum of compound **1**


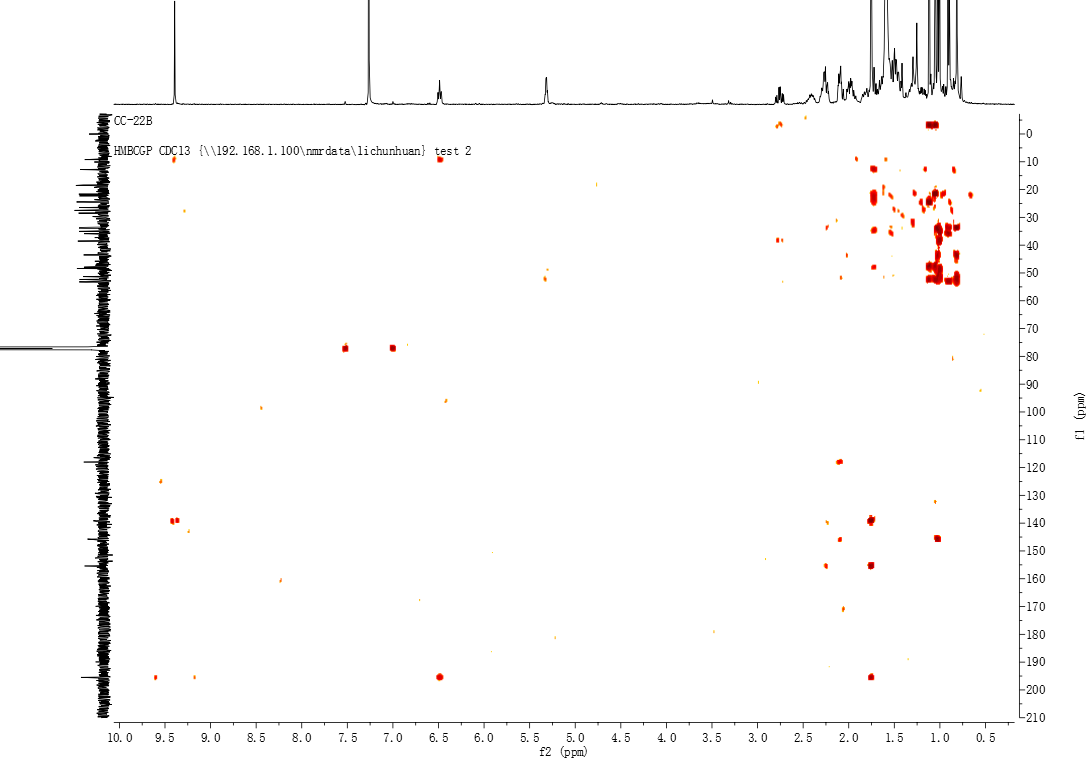


Figure S5. HMBC spectrum of compound **1**


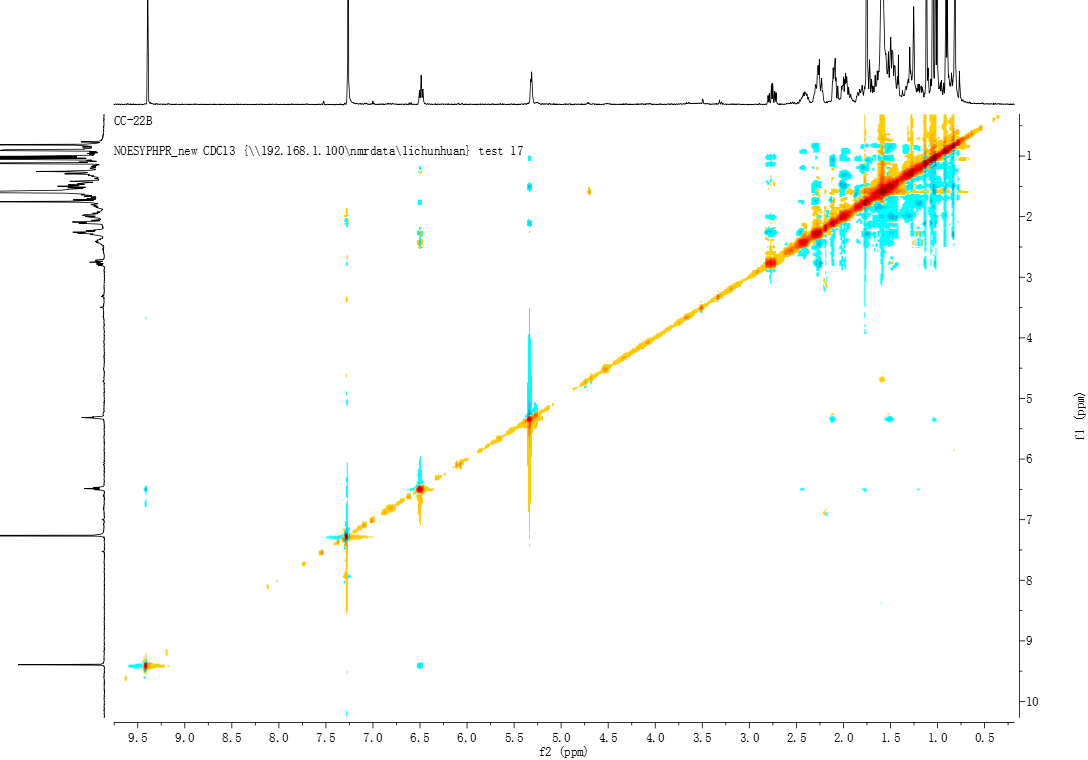


Figure S6. NOESY spectrum of compound **1**

Figure S7. HR-ESI-MS spectrum of compound **1**


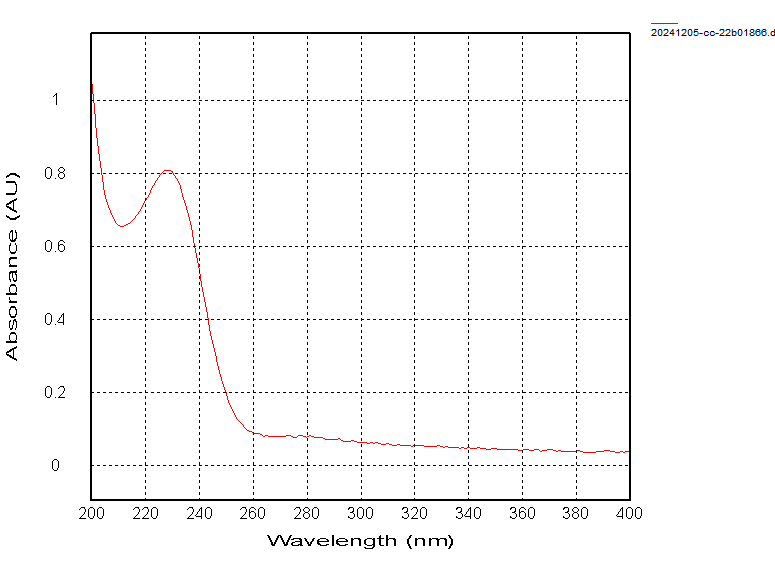


Figure S8. UV spectrum of compound **1** (MeOH)


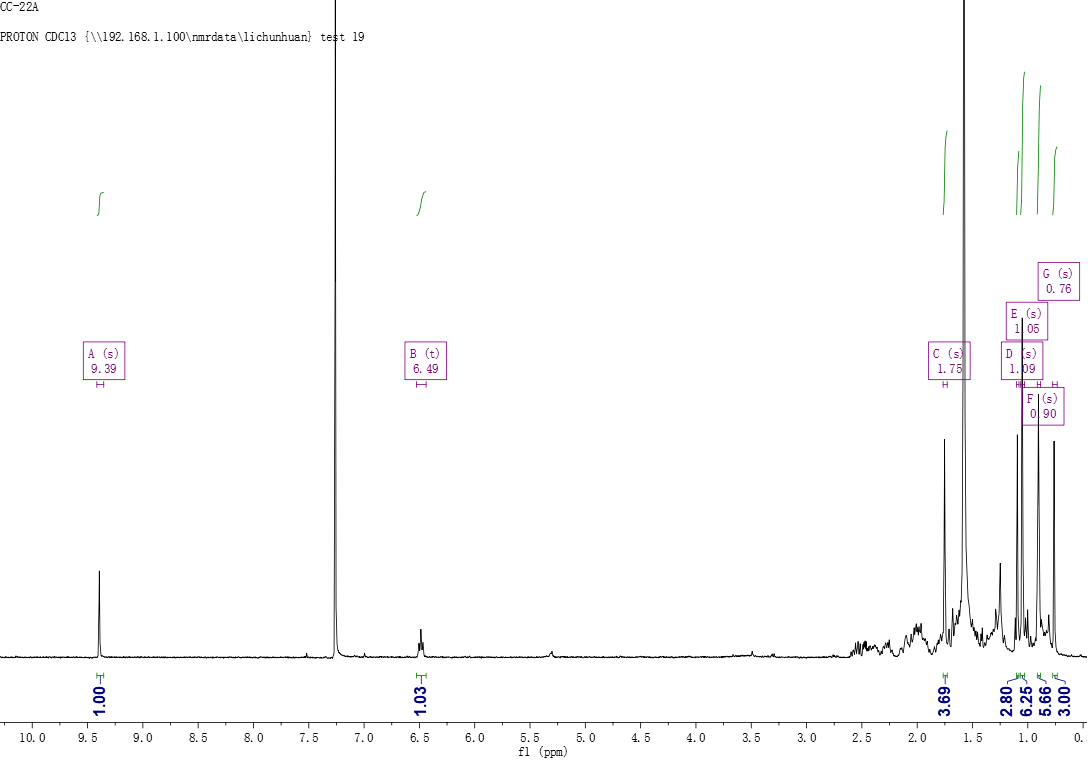


Figure S9. ^1^H NMR spectrum of compound **2** (400MHz, CDCl_3_)


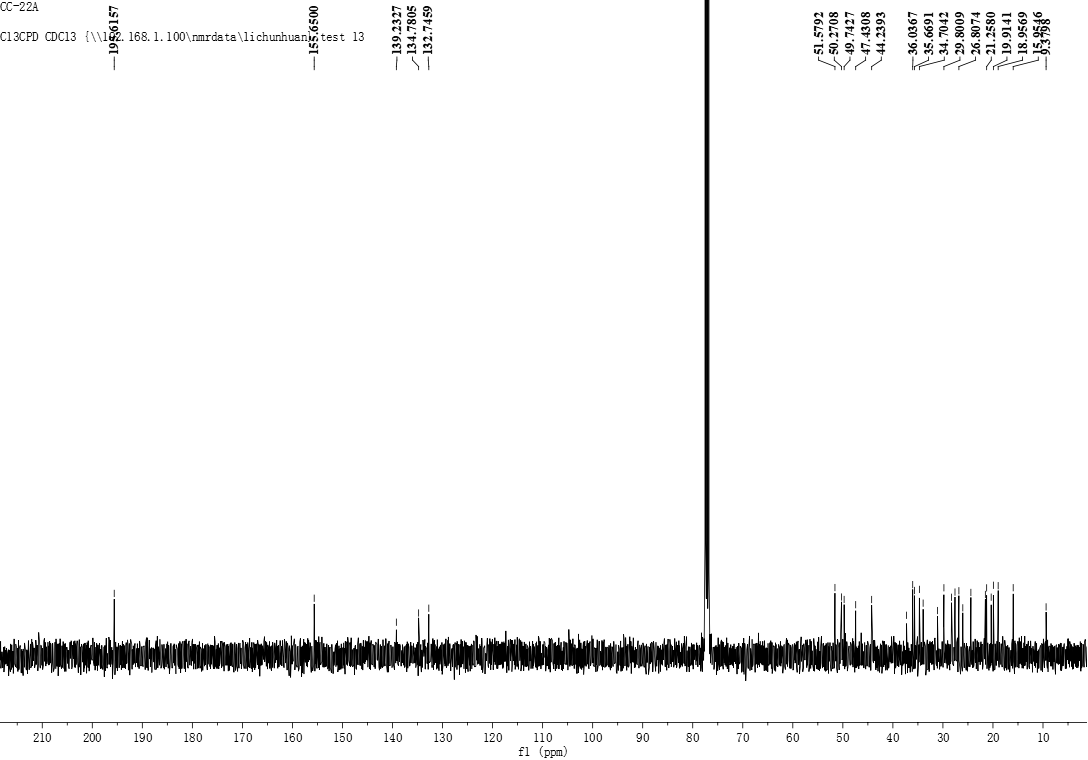


Figure S10. ^13^C NMR spectrum of compound **2** (100MHz, CDCl_3_)


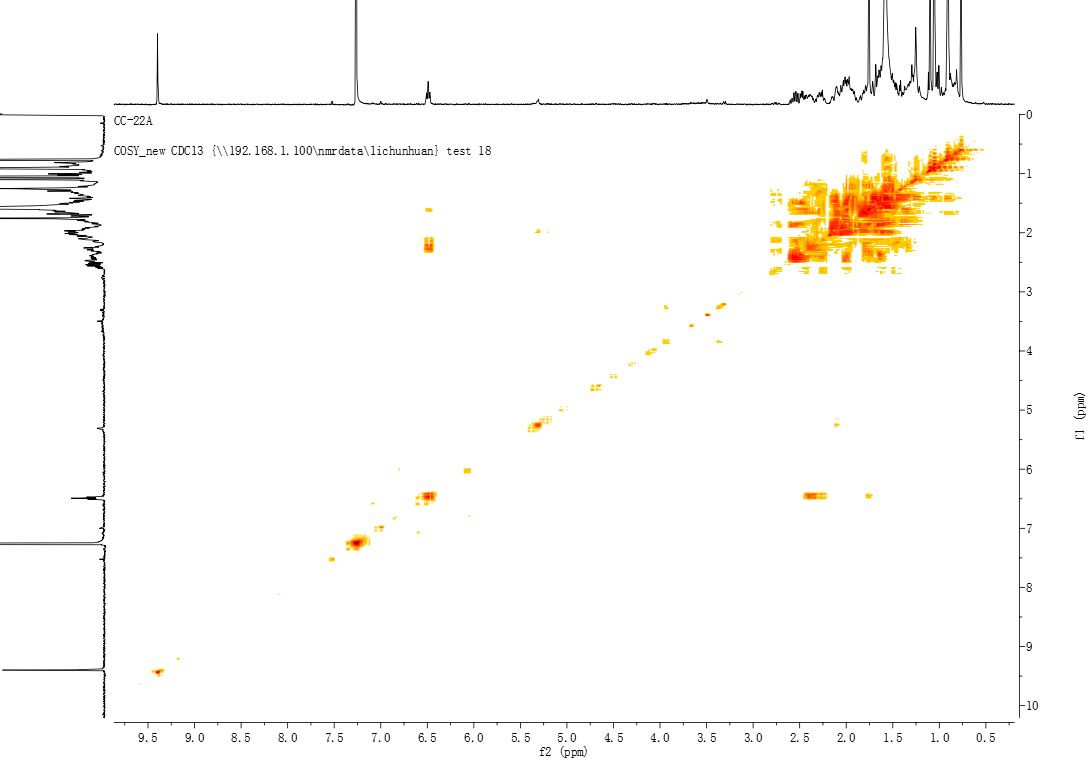


Figure S11. ^1^H-^1^H COSY spectrum of compound **2**


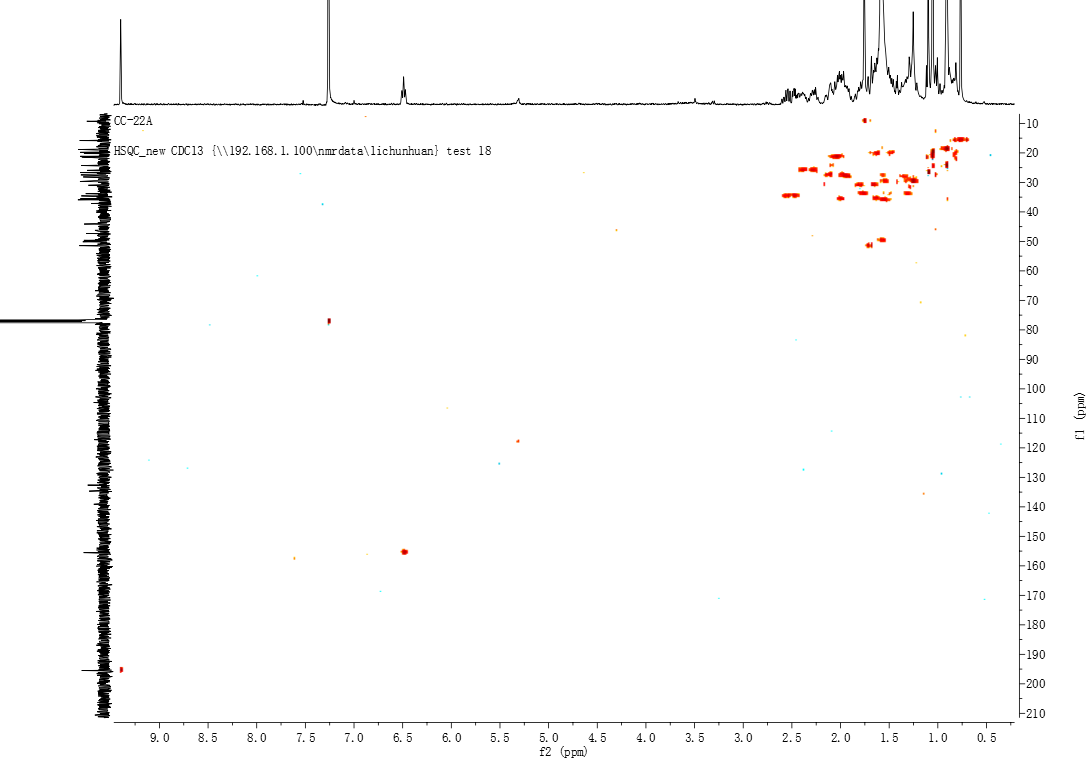


Figure S12. HSQC spectrum of compound **2**


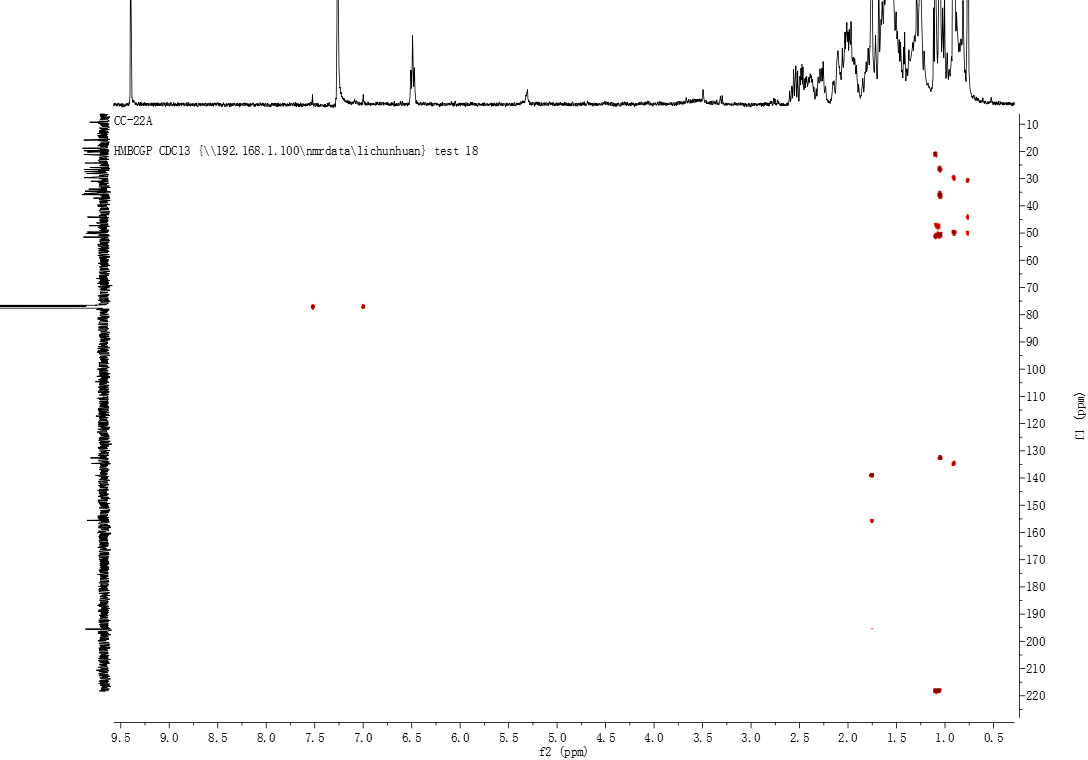


Figure S13. HMBC spectrum of compound **2**


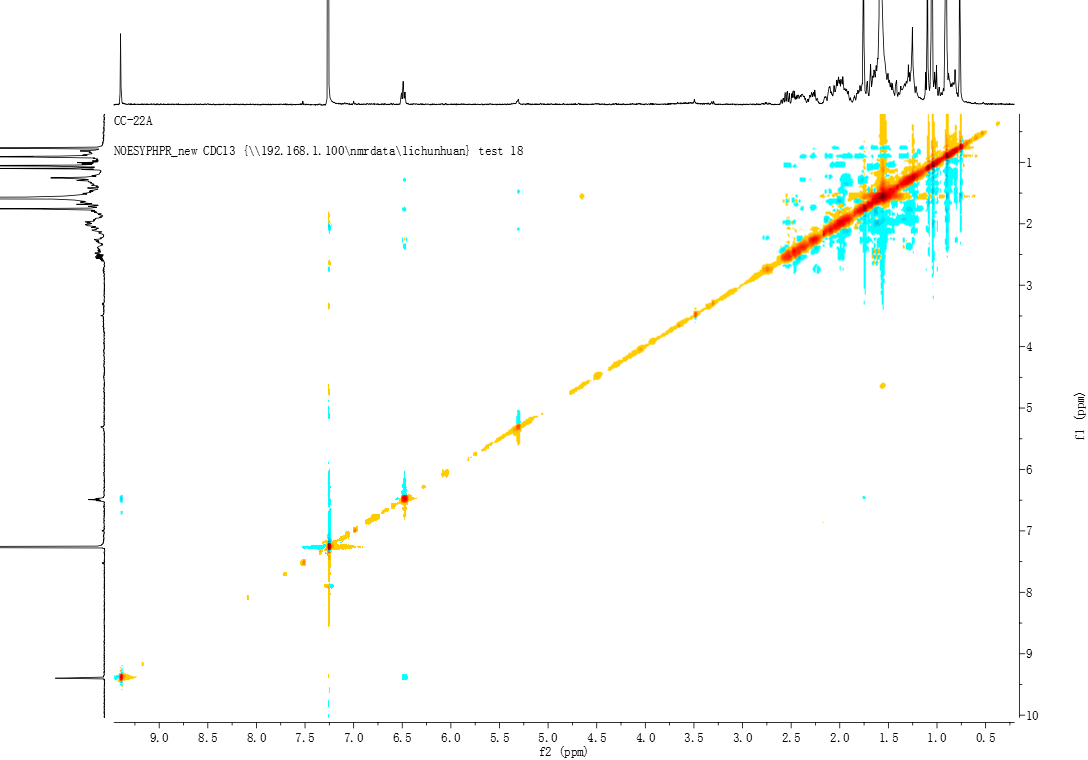


Figure S14. NOESY spectrum of compound **2**

Figure S15. HR-ESI-MS spectrum of compound **2**


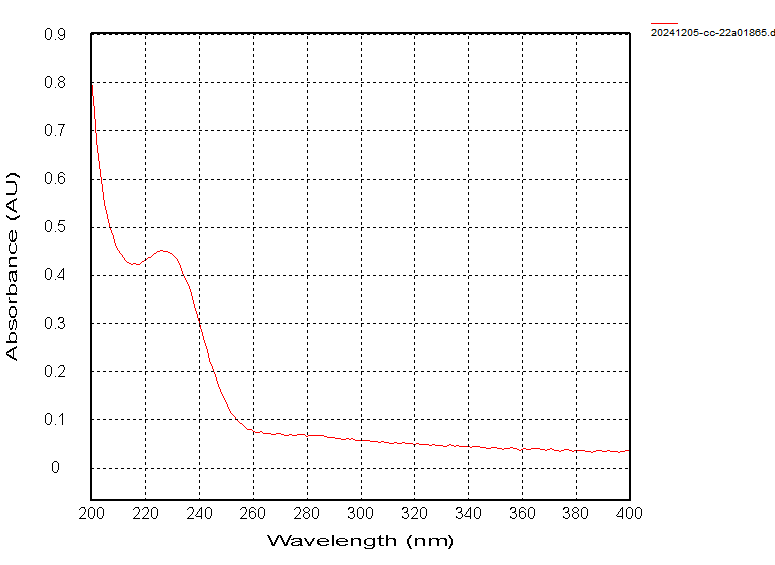


Figure S16. UV spectrum of compound **2** (MeOH)


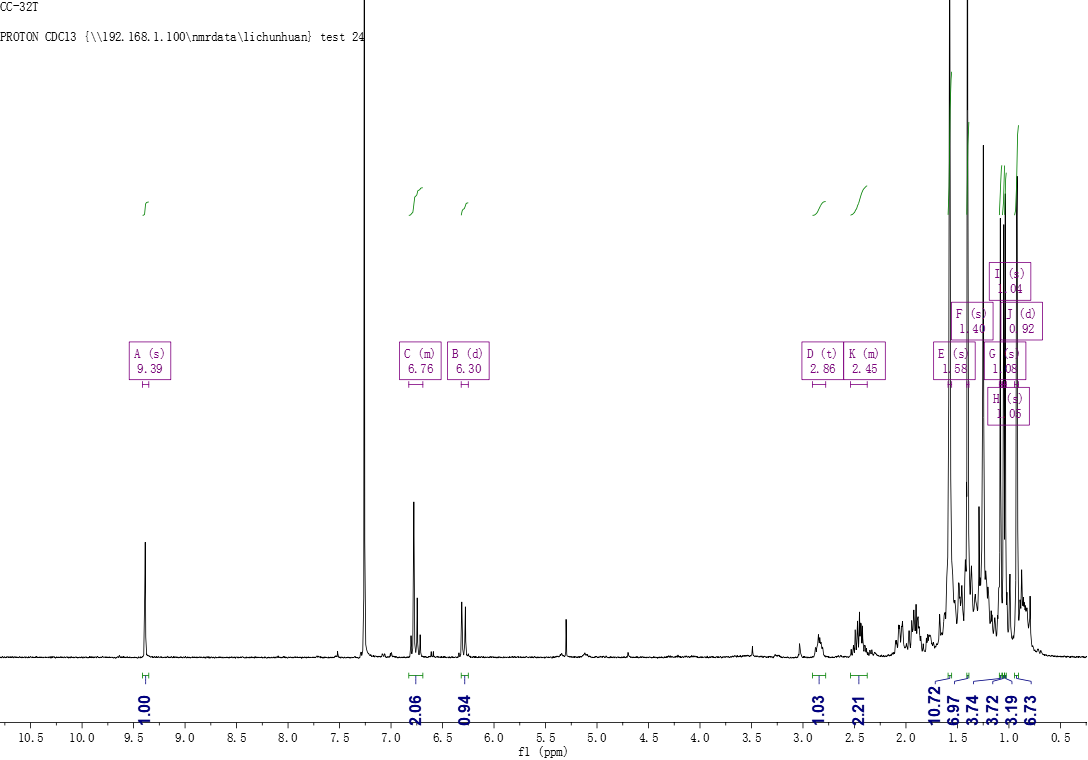


Figure S17. ^1^H NMR spectrum of compound **3** (400MHz, CDCl_3_)


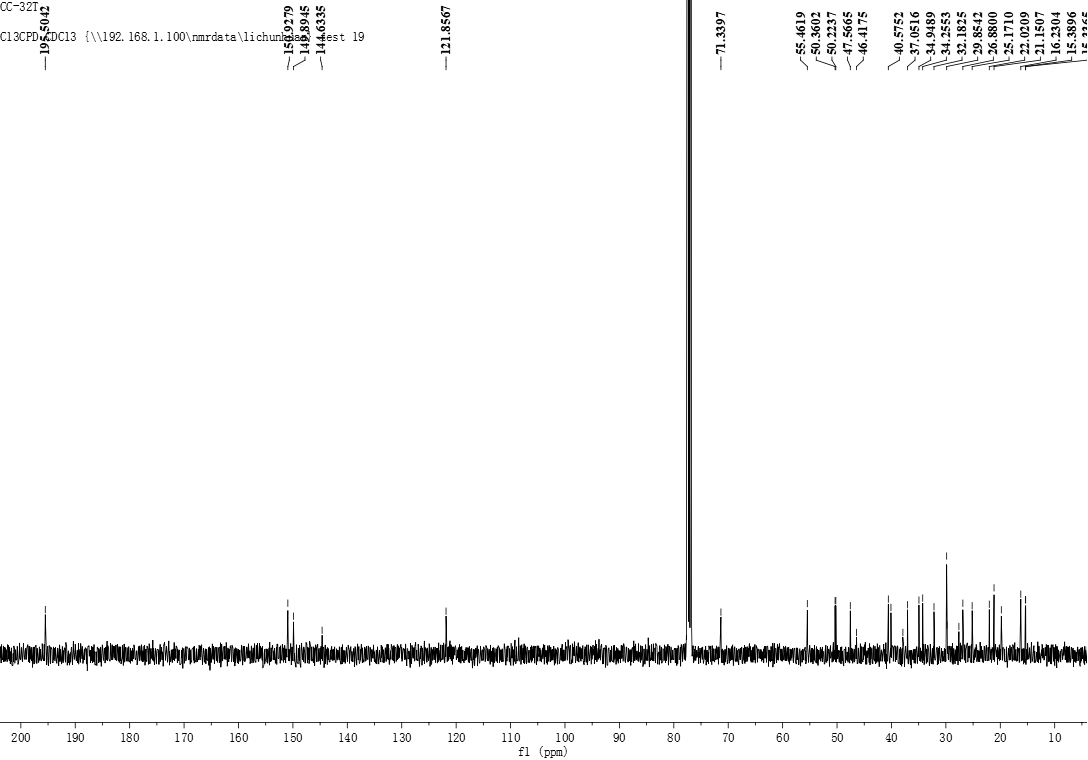


Figure S18. ^13^C NMR spectrum of compound **3** (100MHz, CDCl_3_)


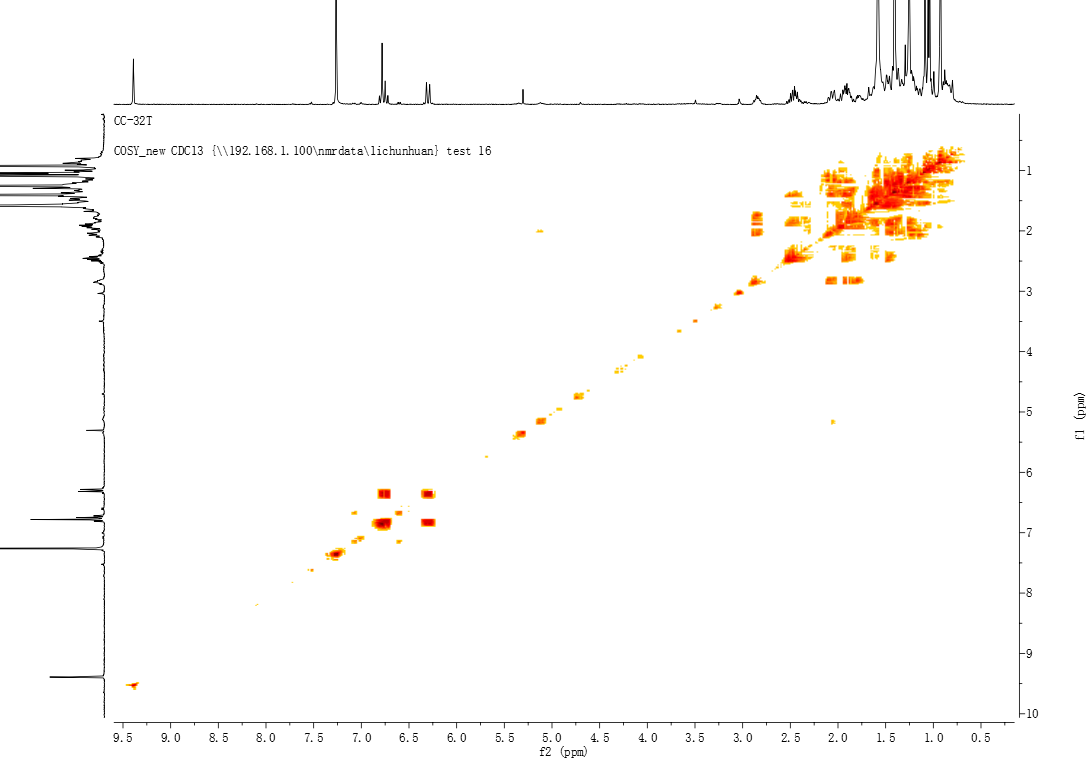


Figure S19. ^1^H-^1^H COSY spectrum of compound **3**


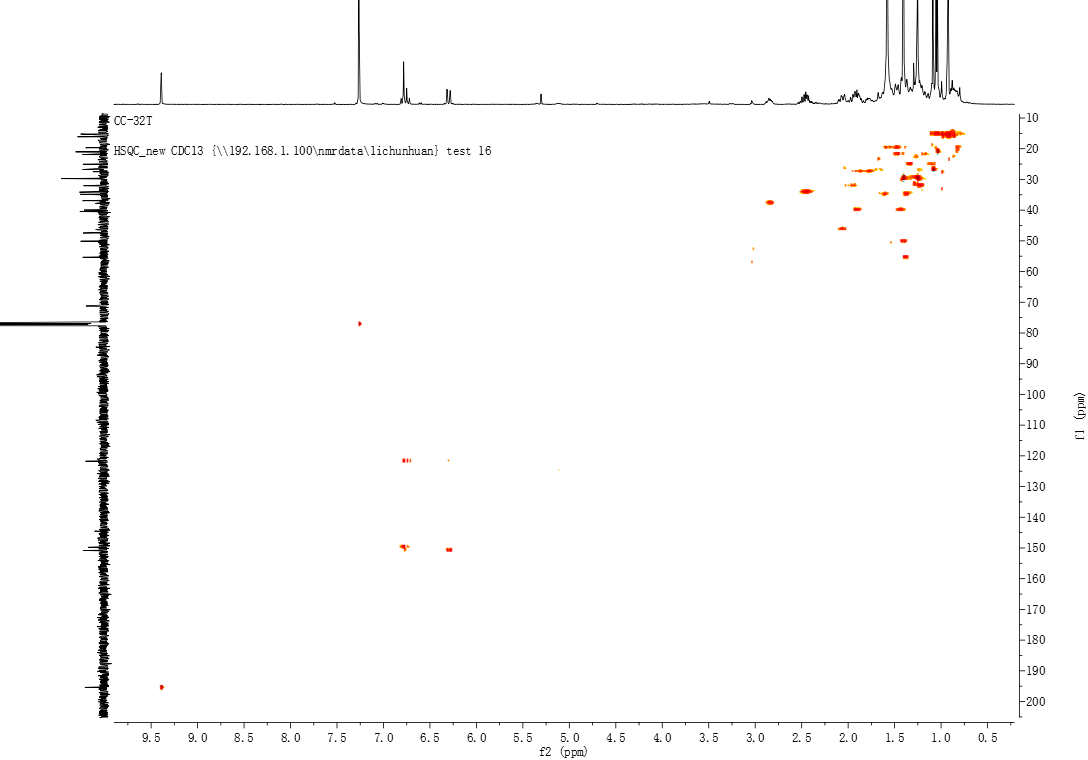


Figure S20. HSQC spectrum of compound **3**


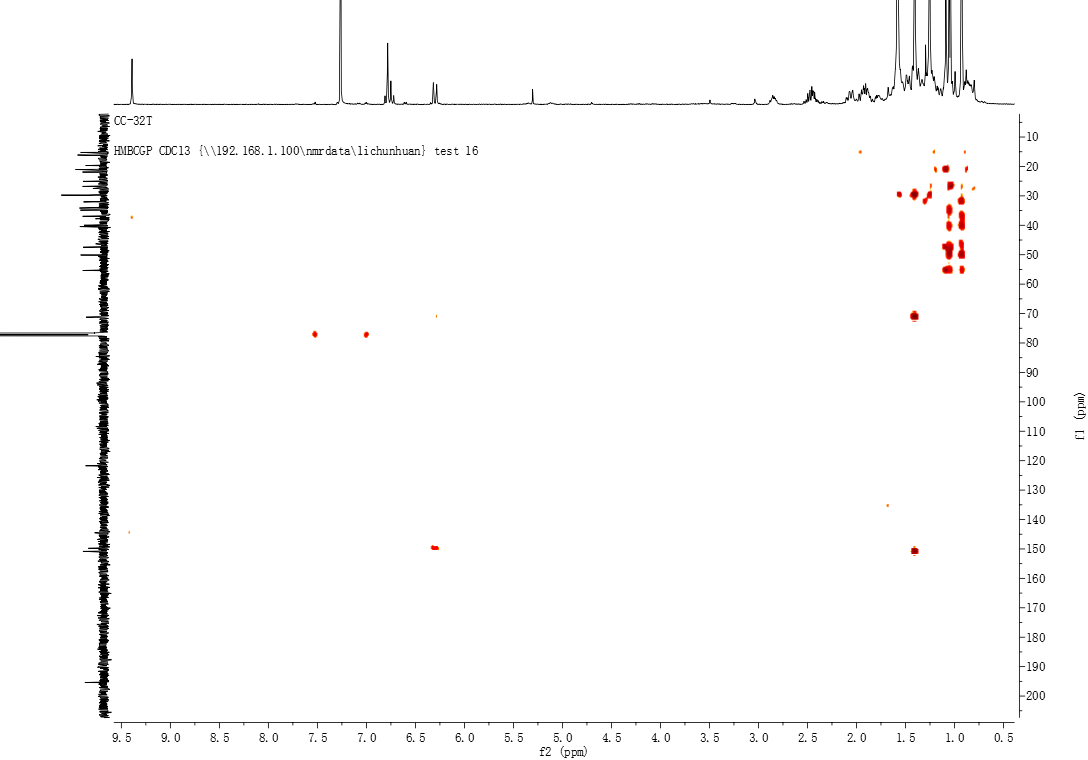


Figure S21. HMBC spectrum of compound **3**


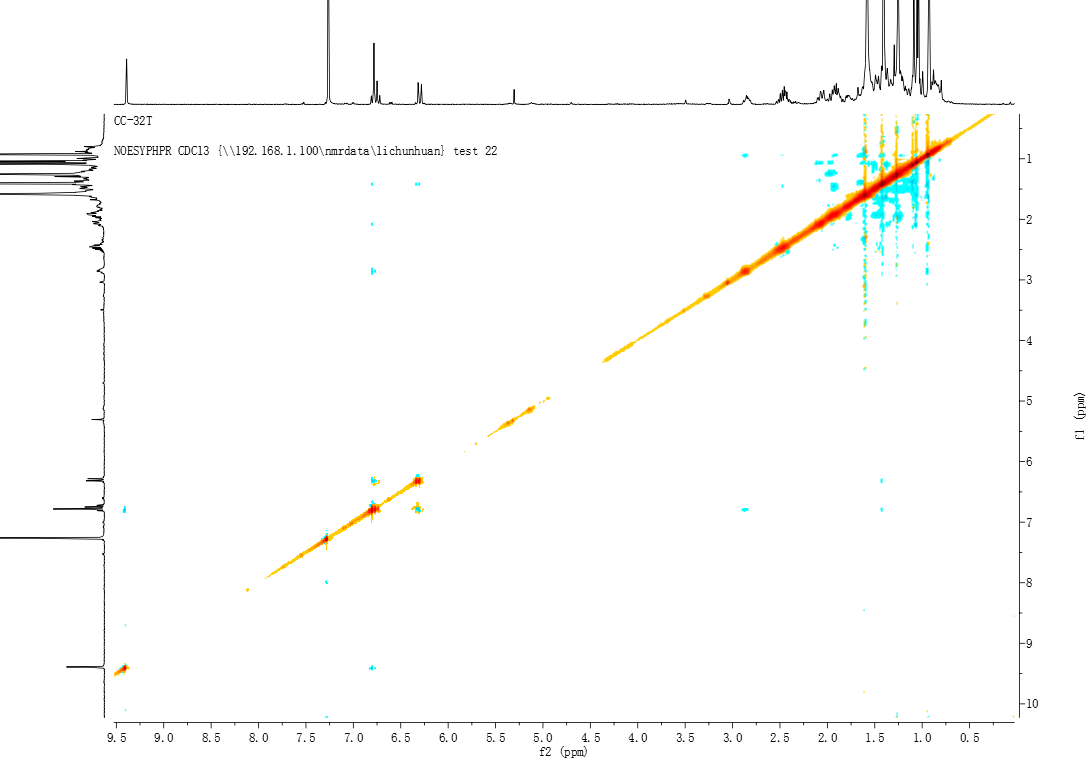


Figure S22. NOESY spectrum of compound **3**

Figure S23. HR-ESI-MS spectrum of compound **3**


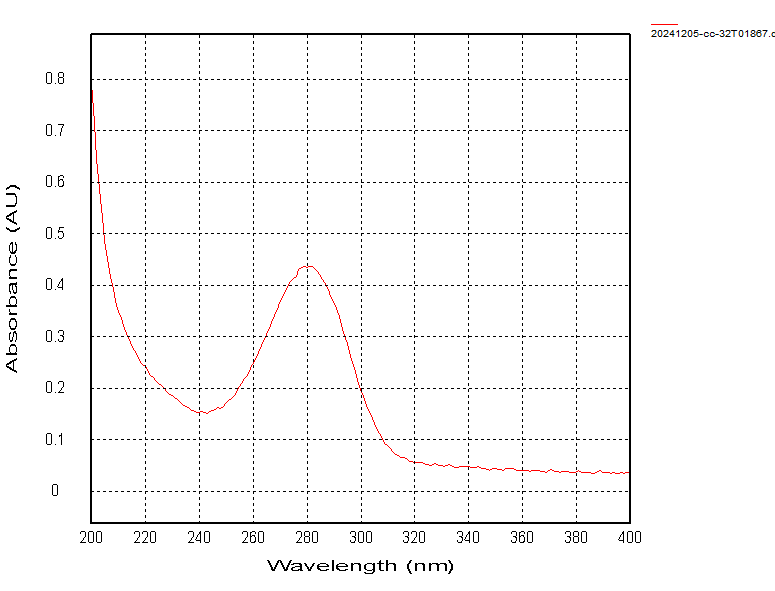


Figure S24. UV spectrum of compound **3** (MeOH)


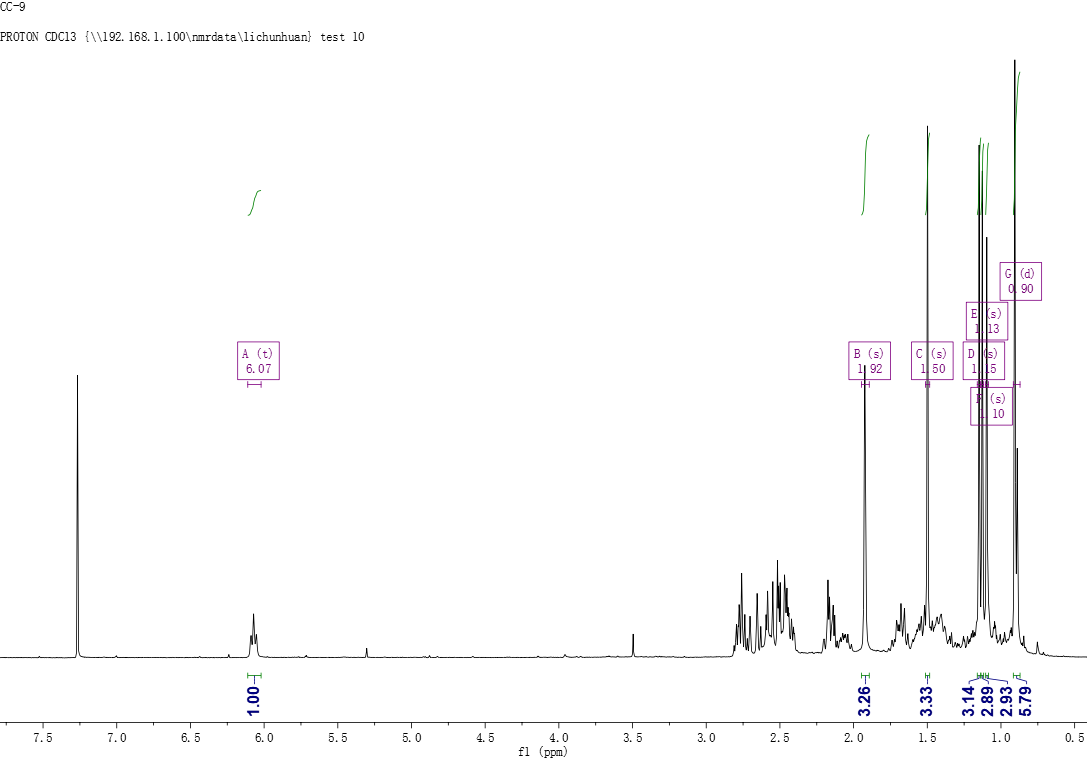


Figure S25. ^1^H NMR spectrum of compound **4** (400MHz, CDCl_3_)


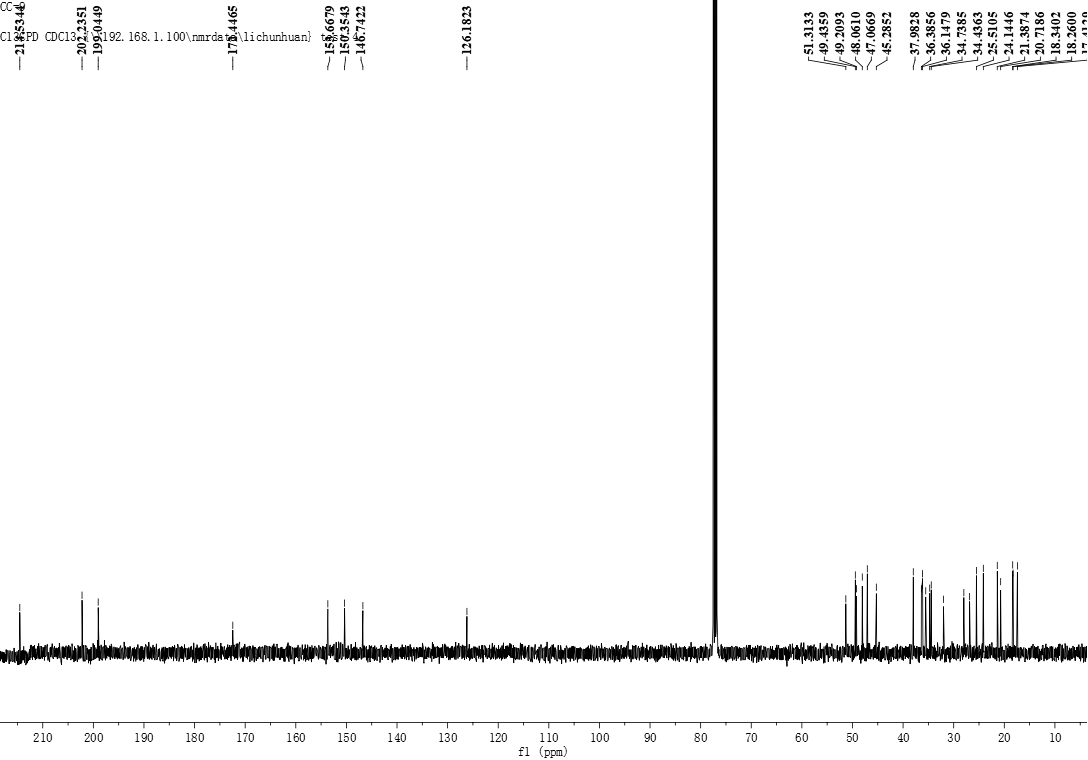


Figure S26. ^13^C NMR spectrum of compound **4** (100MHz, CDCl_3_)


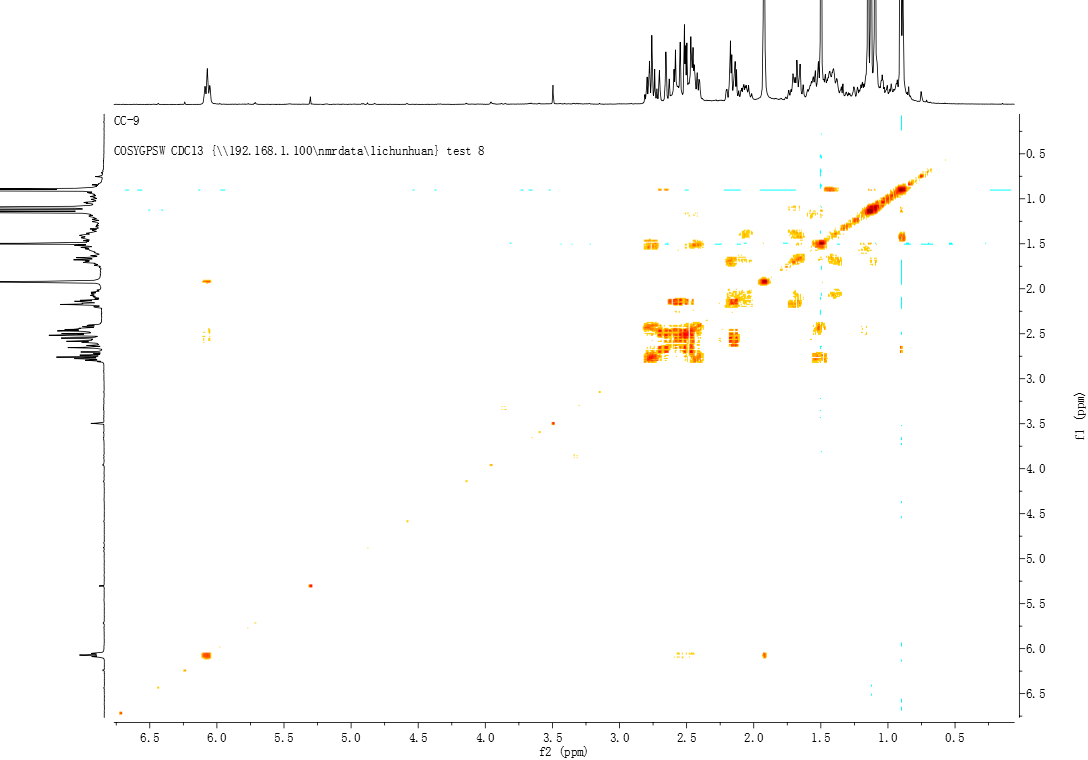


Figure S27. ^1^H-^1^H COSY spectrum of compound **4**


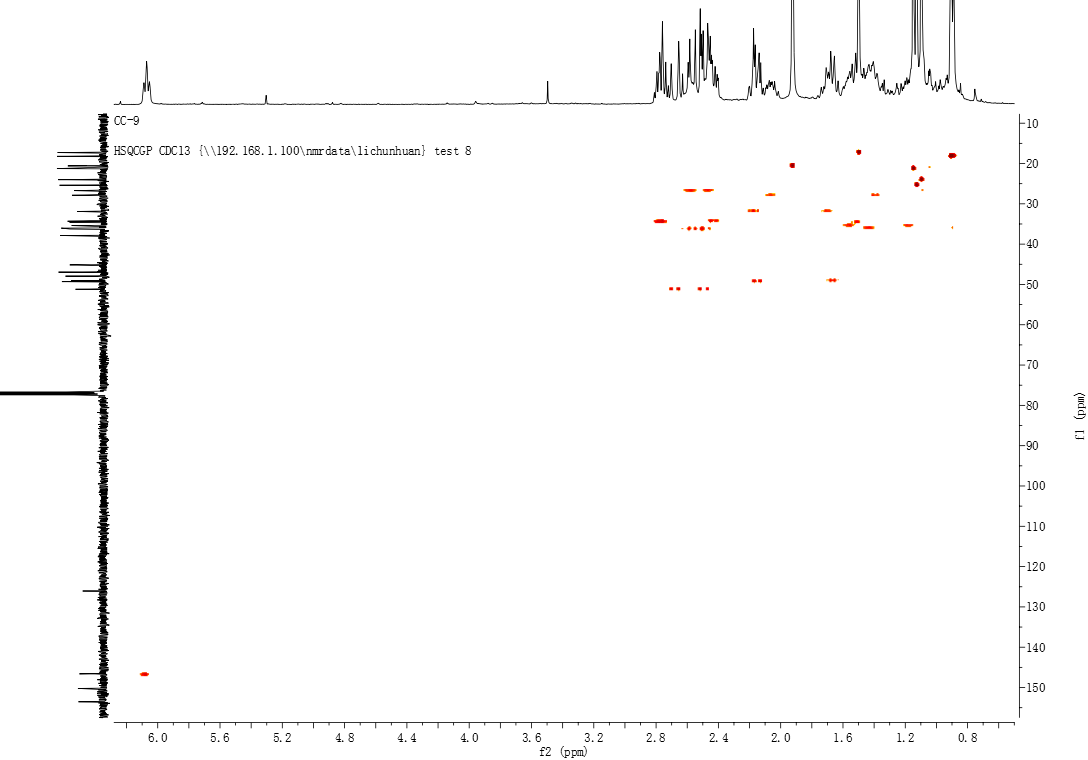


Figure S28. HSQC spectrum of compound **4**


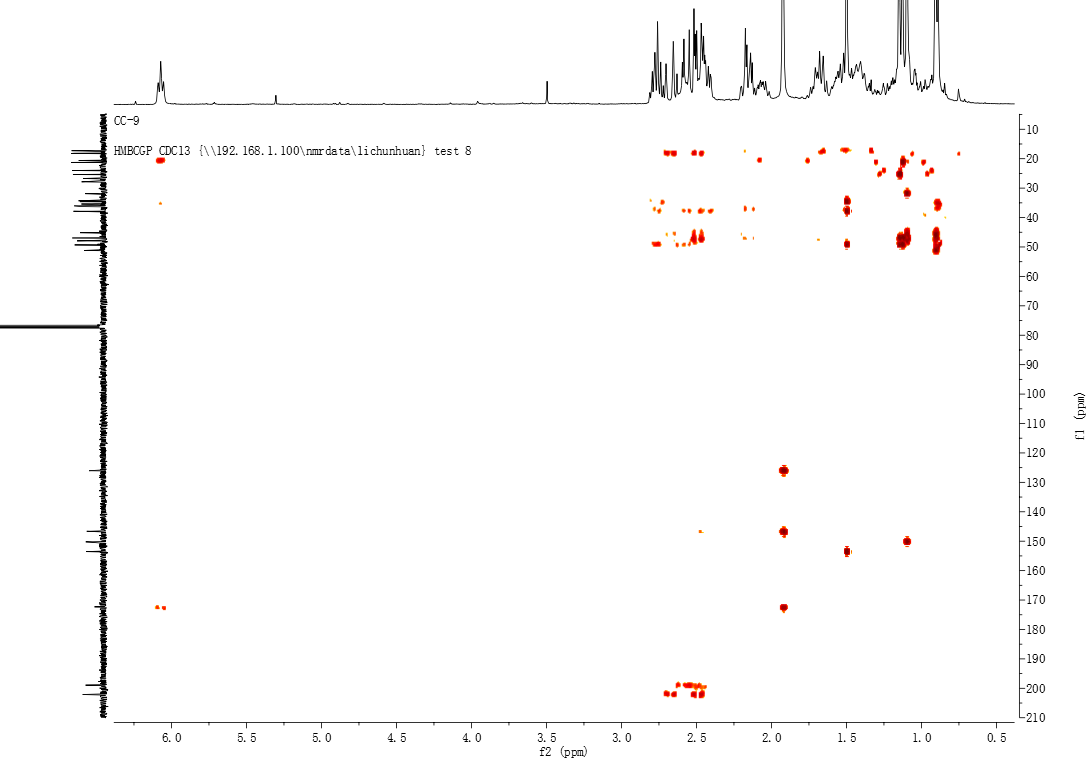


Figure S29. HMBC spectrum of compound **4**


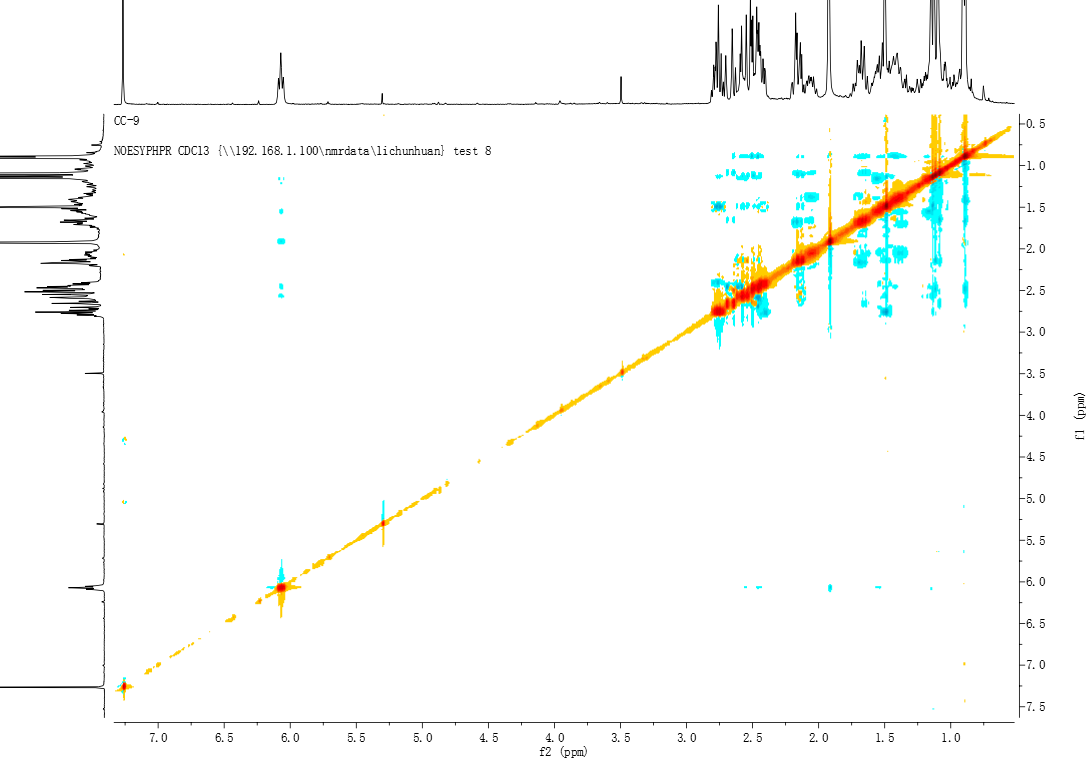


Figure S30. NOESY spectrum of compound **4**

Figure S31. HR-ESI-MS spectrum of compound **4**


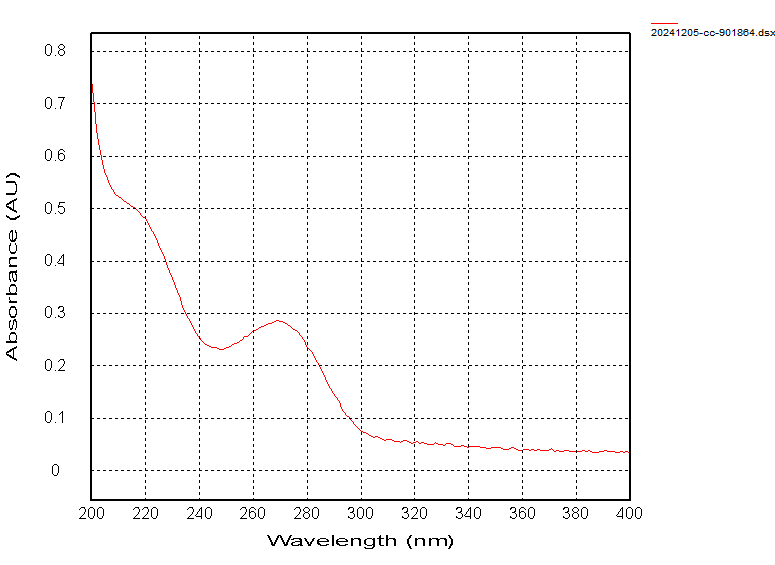


Figure S32. UV spectrum of compound **4** (MeOH)


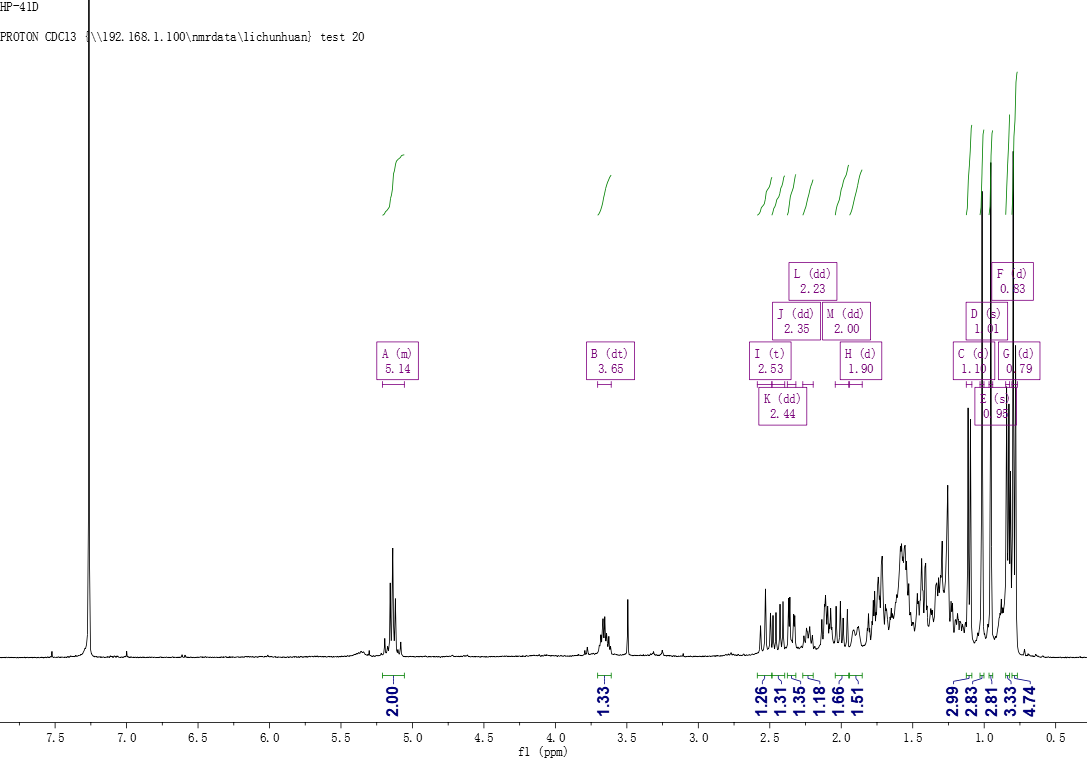


Figure S33. ^1^H NMR spectrum of compound **5** (400MHz, CDCl_3_)


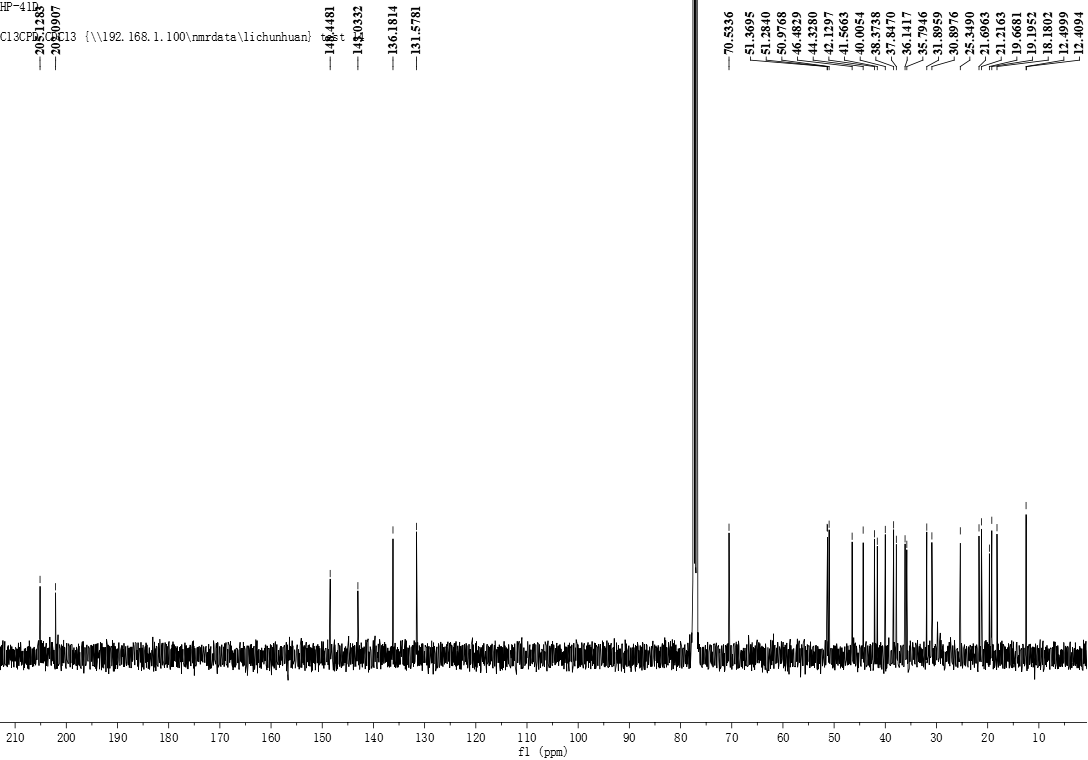


Figure S34. ^13^C NMR spectrum of compound **5** (100MHz, CDCl_3_)


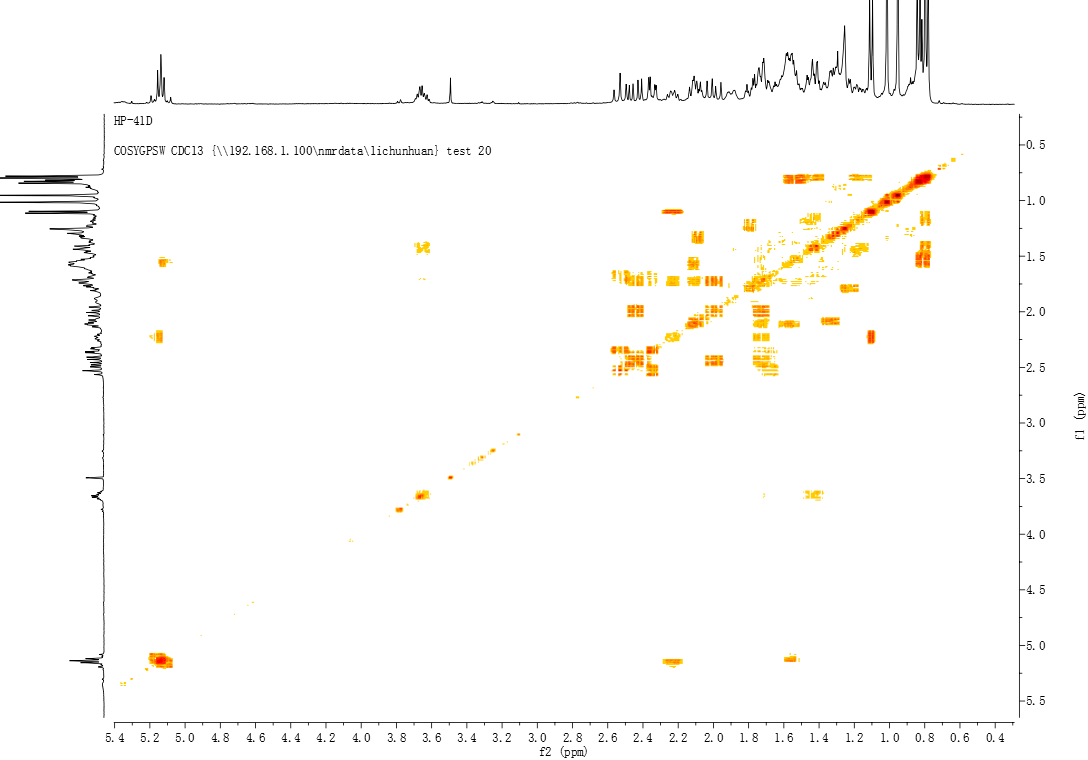


Figure S35. ^1^H-^1^H COSY spectrum of compound **5**


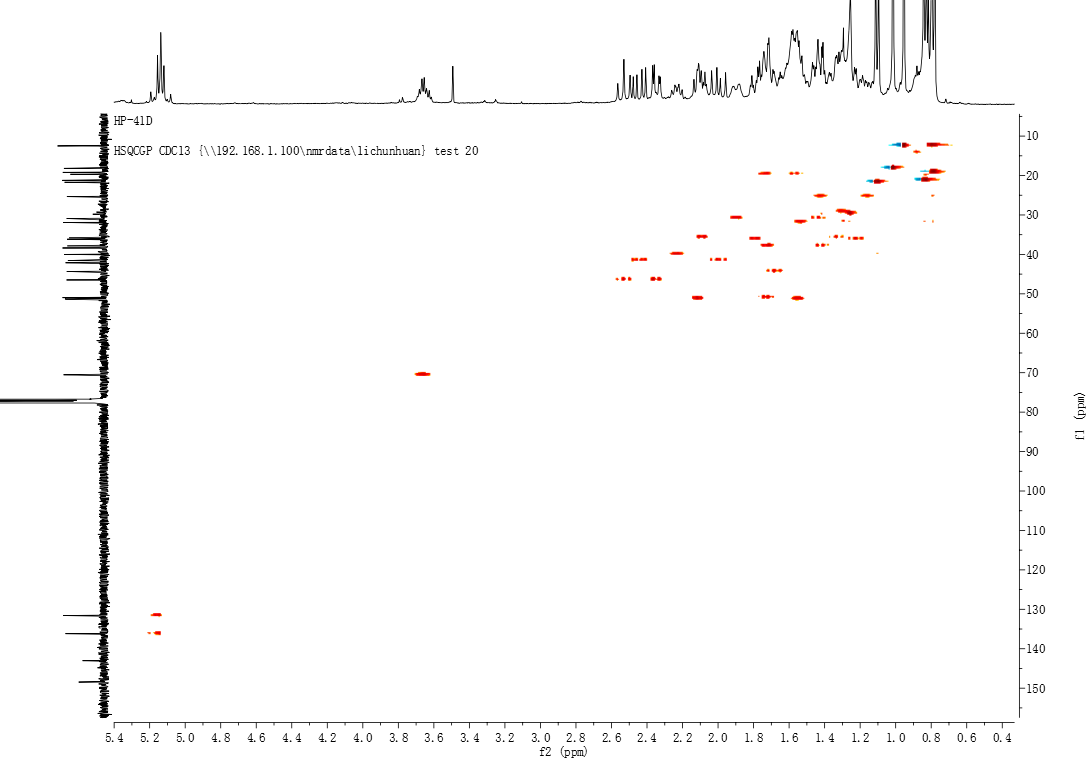


Figure S36. HSQC spectrum of compound **5**


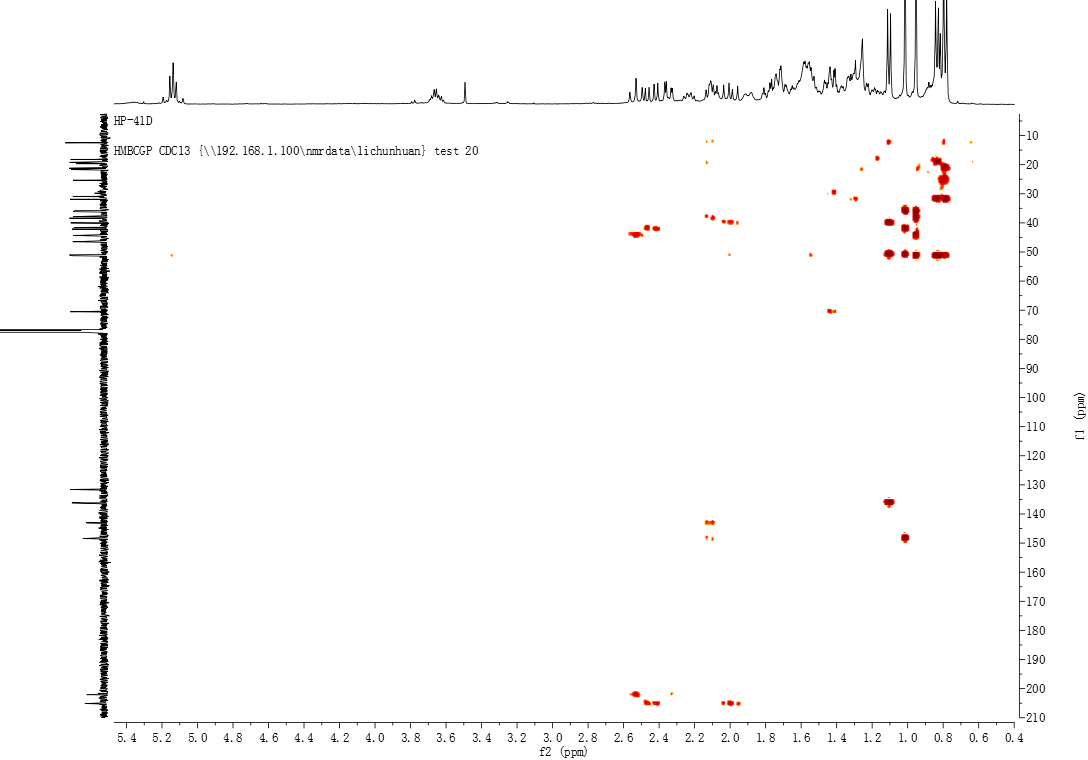


Figure S37. HMBC spectrum of compound **5**


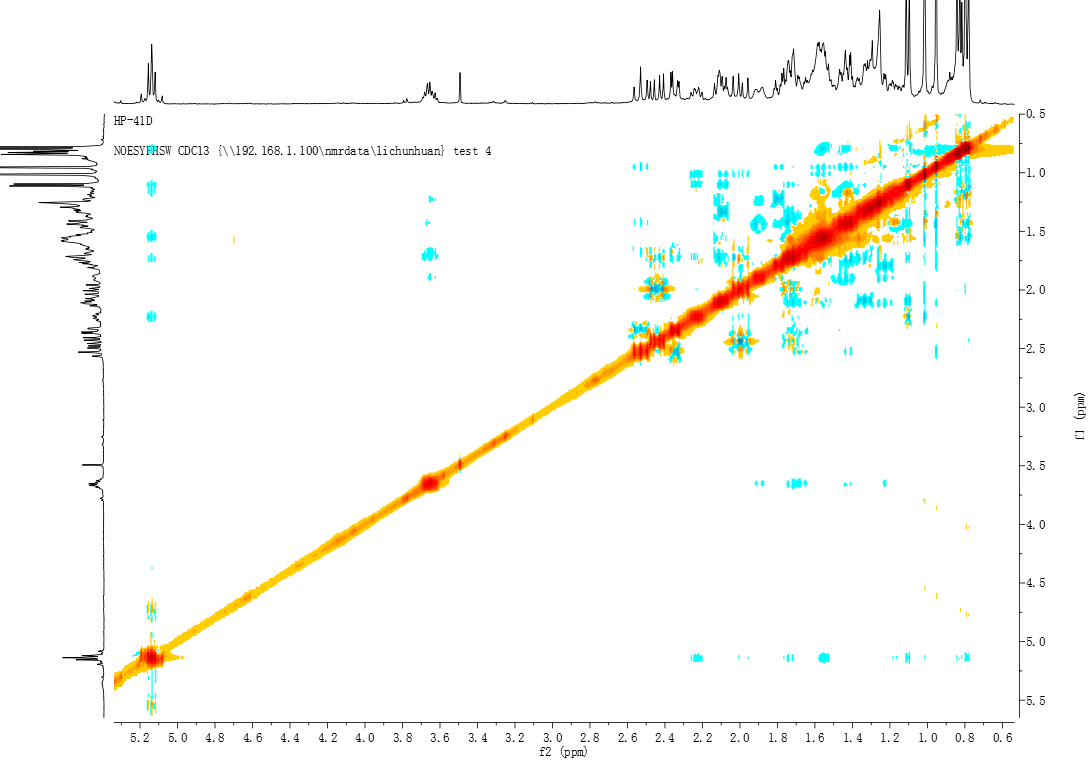


Figure S38. NOESY spectrum of compound **5**

Figure S39. HR-ESI-MS spectrum of compound **5**


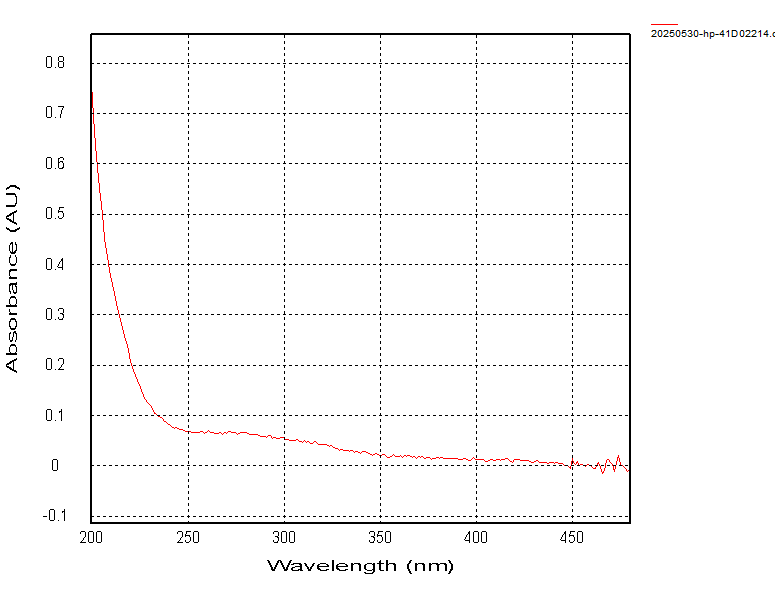


Figure S40. UV spectrum of compound **5** (MeOH)


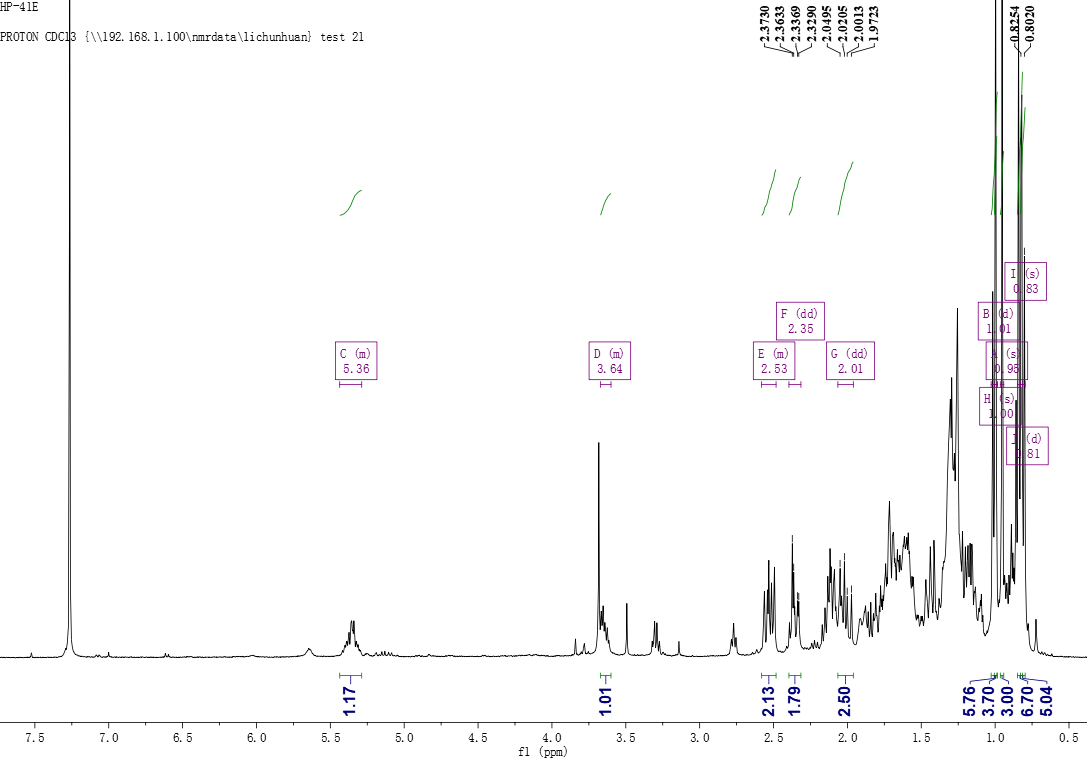


Figure S41. ^1^H NMR spectrum of compound **6** (400MHz, CDCl_3_)


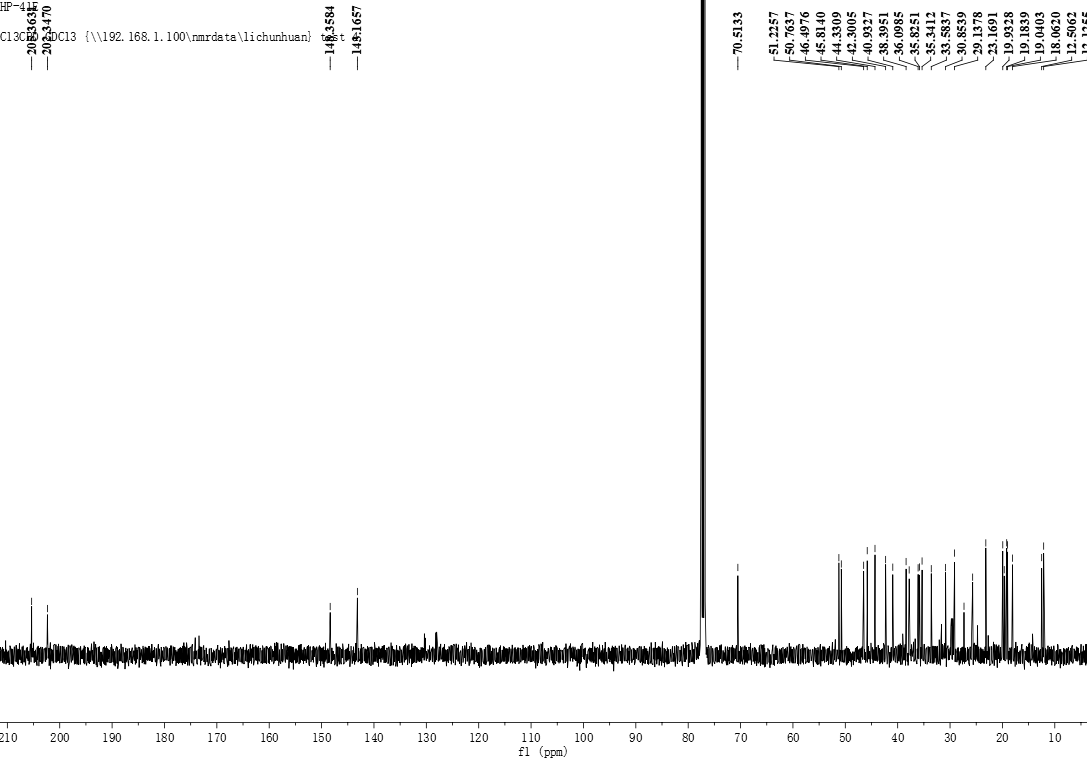


Figure S42. ^13^C NMR spectrum of compound **6** (100MHz, CDCl_3_)


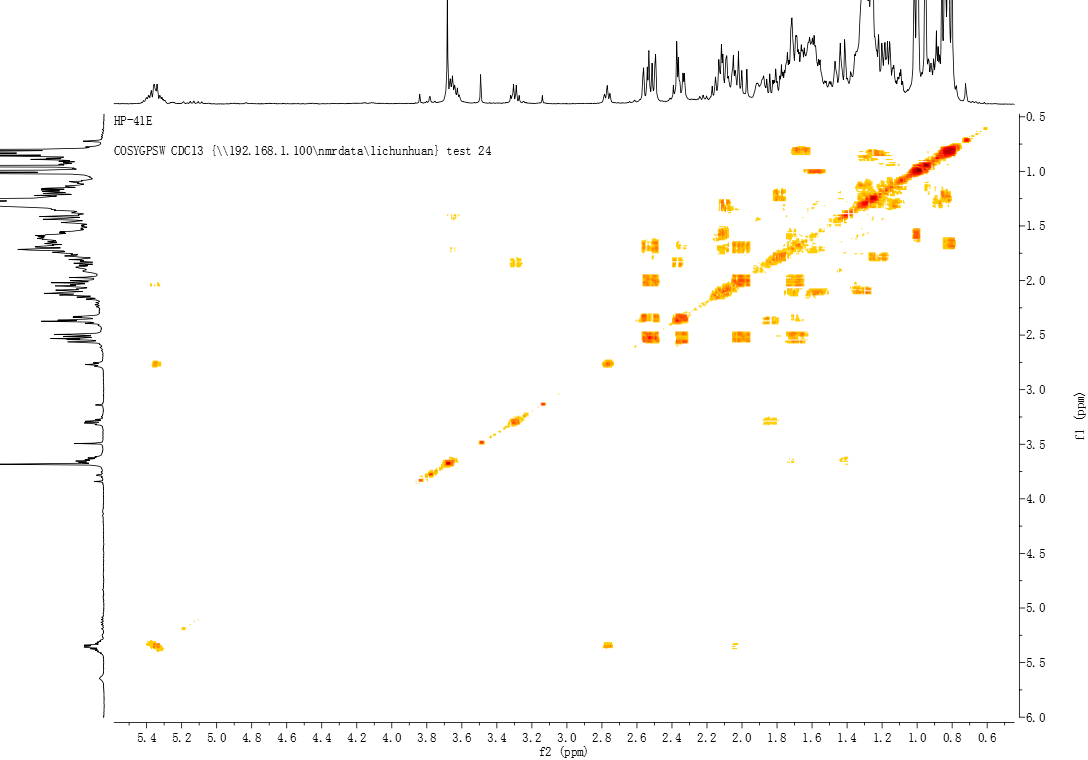


Figure S43. ^1^H-^1^H COSY spectrum of compound **6**


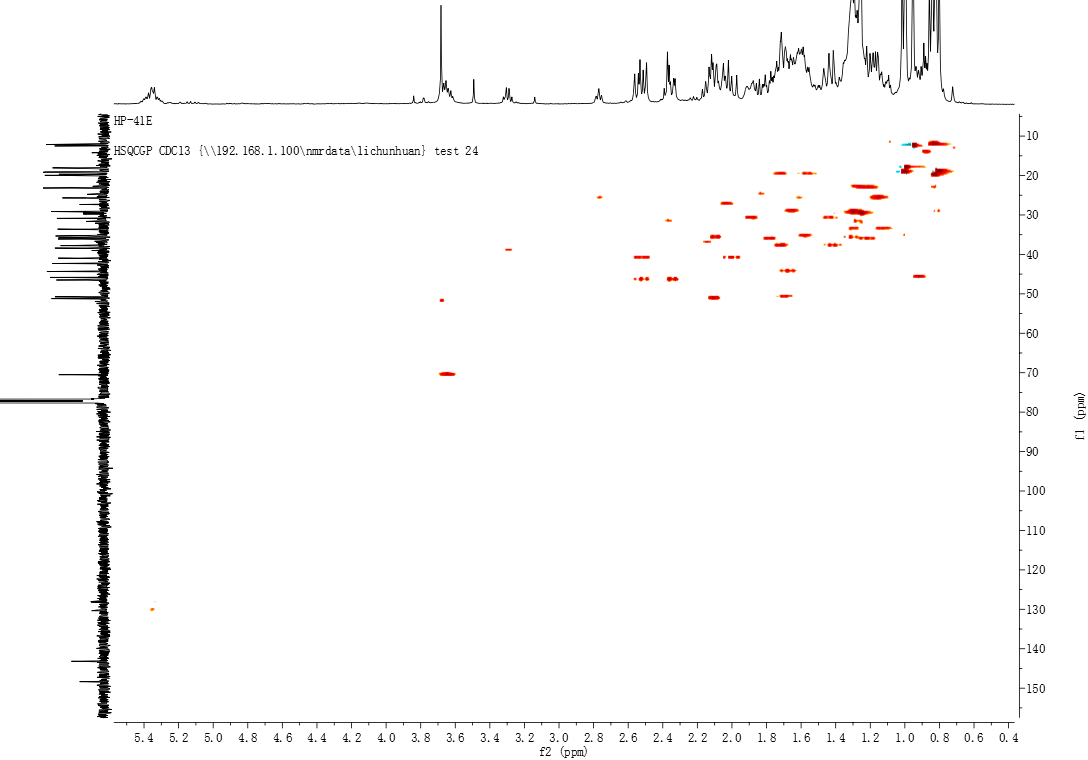


Figure S44. HSQC spectrum of compound **6**


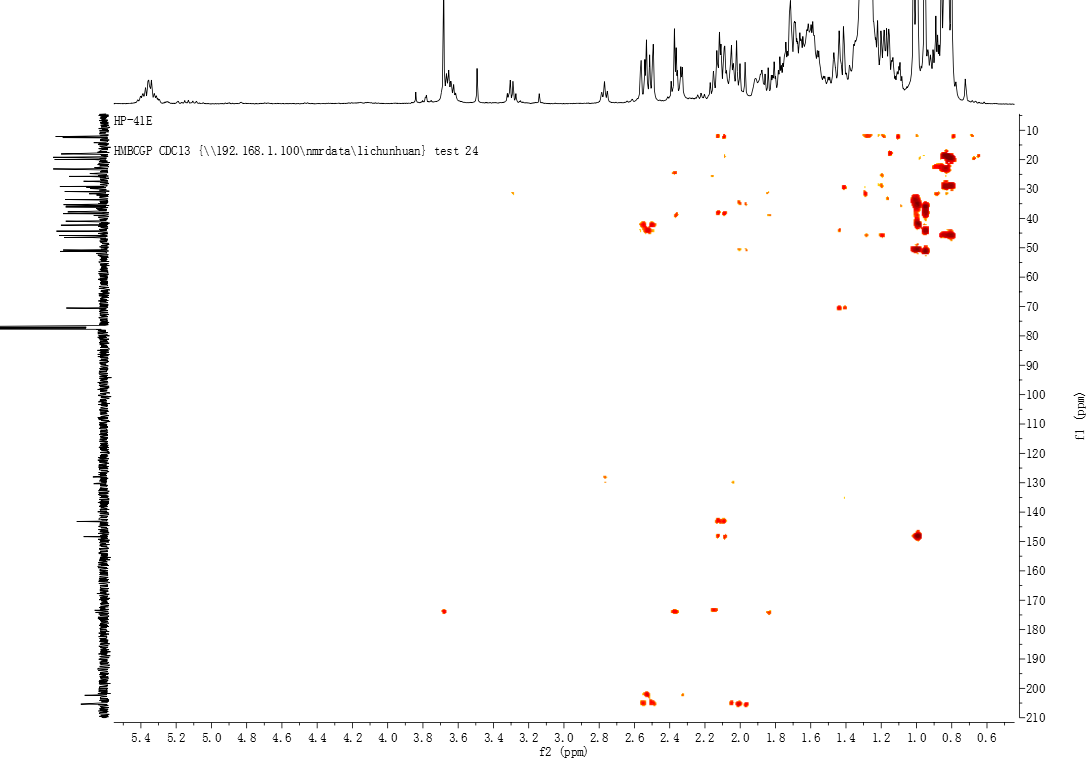


Figure S45. HMBC spectrum of compound **6**


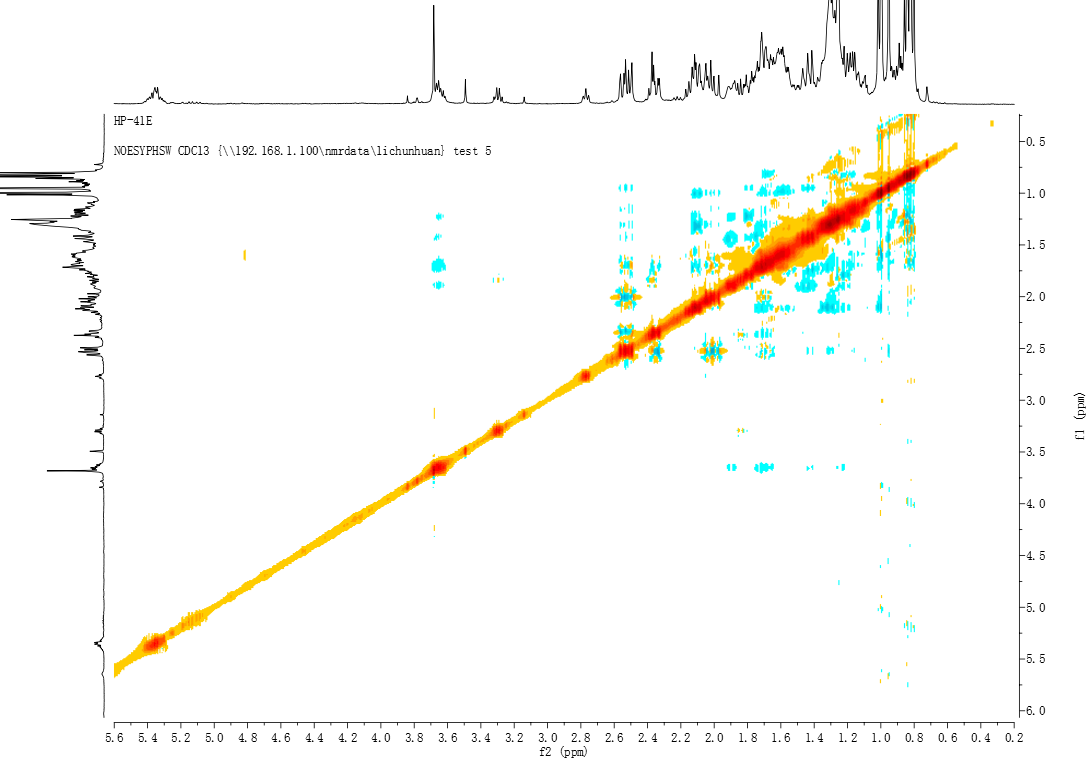


Figure S46. NOESY spectrum of compound **6**

Figure S47. HR-ESI-MS spectrum of compound **6**


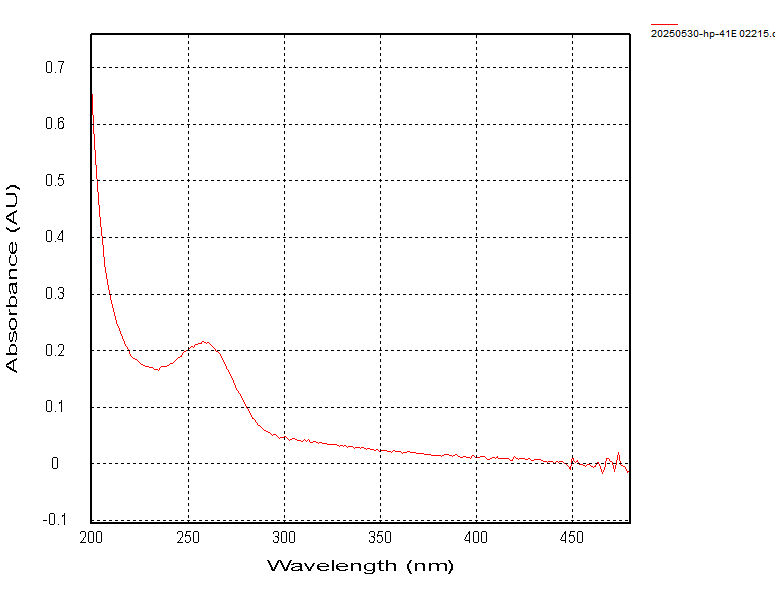


Figure S48. UV spectrum of compound **6** (MeOH)
